# CD47 Knock‐Out Using CRISPR‐Cas9 RNA Lipid Nanocarriers Results in Reduced Mesenchymal Glioblastoma Growth In Vivo

**DOI:** 10.1002/advs.202407262

**Published:** 2025-01-31

**Authors:** Nadia Rouatbi, Adam A. Walters, Alaa Zam, Yau Mun Lim, Alessia Marrocu, Revadee Liam‐Or, Joanne E. Anstee, James N. Arnold, Julie Tzu‐Wen Wang, Steven M. Pollard, Khuloud T. Al‐Jamal

**Affiliations:** ^1^ Institute of Pharmaceutical Science Faculty of Life Sciences and Medicine King's College London Franklin‐Wilkins Building, 150 Stamford Street London SE1 9NH UK; ^2^ Comprehensive Cancer Centre Faculty of Life Sciences and Medicine King's College London, Guy's Hospital London SE1 1UL UK; ^3^ Department of Pharmacology and Pharmacy Li Ka Shing Faculty of Medicine The University of Hong Kong Hong Kong Special Administrative Region China; ^4^ Centre for Regenerative Medicine Institute for Regeneration and Repair & Cancer Research UK Scotland Centre University of Edinburgh 5 Little France Drive Edinburgh EH16 4UU UK; ^5^ Department of Neurodegenerative Disease Queen Square Institute of Neurology University College London London WC1N 3BG UK

**Keywords:** CD47, glioblastoma, immune checkpoint, immunotherapy, nanoparticles, nucleic acid delivery

## Abstract

Immune checkpoint (ICP) blockade has shown limited effectiveness in glioblastoma (GBM), particularly in the mesenchymal subtype, where interactions between immune cells and glioblastoma cancer stem cells (GSCs) drive immunosuppression and therapy resistance. Tailoring ICPs specific to GSCs can enhance the antitumor immune response. This study proposes the use of lipid nanoparticles (LNPs) encapsulating CRISPR RNAs as an in vivo screening tool for ICPs in a syngeneic model of mesenchymal GSCs. Using PD‐L1 and CD47 to validate the proof of concept, intratumoral administration of LNPs in orthotopic tumors achieved efficient editing of ICPs, leading to enhanced immune cell infiltration within the tumor microenvironment. Targeting CD47 reduced tumor growth, suggesting improved cancer cell sensitization to the immune system post‐ICP editing. The study positions LNPs as a robust tool for in vivo validation of ICPs as therapeutic targets in clinically relevant GBM models. LNPs could serve as a screening tool in patient‐derived xenografts to identify and optimize ICP combinations, potentially expediting ICP translation and enhancing personalized GBM immunotherapies.

## Graphical abstract

Lipid nanoparticles encapsulating CRISPR RNAs are used as an in vivo screening tool to identify immune checkpoint targets in glioblastoma. In a mesenchymal glioblastoma model, CD47 knockout via lipid nanoparticles enhances immune cell infiltration and reduces tumor growth. This approach enables rapid validation of immune checkpoint therapies and supports the development of personalized GBM immunotherapies.

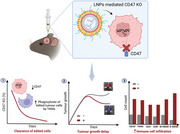

## 1 Introduction

Glioblastoma Multiforme (GBMrepresents one of the most aggressive malignant brain tumors. The prevailing standard of care encompasses maximal surgical resection followed by concurrent chemotherapy and radiotherapy. However, despite this aggressive approach, ≈80% of patients suffer GBM recurrence, marked by a heightened degree of infiltration and aggressiveness compared to primary tumors.^[^ ^1^ ^]^ GBM can be categorized into four subtypes based on its molecular signature: proneural, neural, classical, and mesenchymal.^[^ ^2^ ^]^ Among these, mesenchymal tumors exhibit a marked aggressiveness characterized by high angiogenesis, hypoxia, necrosis, and invasiveness.^[^ ^3^ ^]^ Treatment options for mesenchymal GBM are further restricted as patients display a limited response to radiation therapy.^[^ ^3^ ^]^ Unfortunately, it has been observed that, following treatment, some GBM subtypes tend to undergo transcriptional shifts toward a mesenchymal phenotype.^[^ ^3^, ^4^ ^]^

The mesenchymal tumor microenvironment (TME) is marked by an elevated immune cell infiltration.^[^ ^5^ ^]^ Immune cells can comprise >50% of the tumor bulk, with tumor‐associated macrophages/microglia (TAMs) constituting the majority of infiltrating immune cells.^[^ ^6^ ^]^ Within this complex milieu, reciprocal interactions between TAMs and GBM cells trigger a cascade of signaling pathways that foster an immunosuppressive microenvironment. This dynamic crosstalk between immune and cancer cells supports the plasticity and adaptability of GBM, promoting heterogeneity, invasiveness, and immune suppression, thereby intensifying disease progression.^[^ ^7^ ^]^ The substantial immune cell infiltration renders GBM a promising candidate for cancer immunotherapy.

The past two decades have borne witness to remarkable progress in cancer treatment, particularly with the introduction of cancer immunotherapies. Designed to activate the immune system against cancer cells, one such approach is immune checkpoint blockade. Immune checkpoints (ICPs) encompass immune‐stimulatory and suppressive molecules responsible for maintaining immune system equilibrium. In the context of malignancies, this balance is disrupted, impeding the immune system's ability to recognize and eliminate cancer cells, thereby fostering tumor growth. ICPs such as cluster of differentiation 47 (CD47) and programmed death ligand 1 (PD‐L1), frequently overexpressed by cancer cells, play critical roles. CD47 functions as a “do not eat me” signal, binding to its receptor signal‐regulatory protein alpha (SIRP α) on macrophages and microglia, impeding their phagocytic capacity.^[^ ^8^ ^]^ PD‐L1 interacts with PD‐1 expressed in T cells, blocking the activation of antigen‐specific effector T cells.^[^ ^9^ ^]^ Since 2010, multiple monoclonal antibodies targeting different ICPs (e.g., PD‐1, PD‐L1, CTLA‐4, CD47) have been developed^[^ ^10^ ^]^ and are now integrated into the standard of care for treating melanoma, non‐small cell lung cancer, and other solid tumors.^[^ ^11^, ^12^ ^]^

Immunotherapy has exhibited promise against GBM in preclinical studies.^[^ ^13^, ^14^ ^]^ Nonetheless, despite favorable safety outcomes in various clinical trials, these have fallen short of yielding significant survival benefits during phase III studies.^[^ ^15^, ^16^ ^]^ Challenges that impede the efficacy of such therapies encompass the blood‐brain barrier, which restricts the brain's permeability to therapeutic agents.^[^ ^17^ ^]^ The high incidence of pro‐tumorigenic TAMs and the reduced T‐cell infiltration limit the efficacy of immune checkpoint blockade in generating durable immune responses against GBM.^[^ ^18^, ^19^ ^]^ Additionally, GBM cancer stem cells (GSCs) directly interact with the TME employing various strategies to evade the immune system. GSCs can recruit and activate myeloid‐derived suppressor cells (MDSCs), promote the expansion of immunosuppressive TAMs, and inhibit the activation of T cells. Furthermore, GSCs can adjust to different stressors, such as therapies and metabolic changes, promoting the clonal expansion of new resistant clones, resulting in increased heterogeneity and treatment resistance.^[^ ^20^ ^]^ To add to the disease complexity, current mouse models fail to mirror the intricate immunosuppressive TME and the intra‐ and inter‐tumoral variabilities observed clinically.^[^ ^21^ ^]^ As such there is a notable translational gap between preclinical observations and the clinical reality, necessitating the need for more representative models and tools to test hypotheses and validate novel therapeutic targets.

The advent of clustered regularly interspaced short palindromic repeats (CRISPR)‐associated protein 9 (CRISPR‐Cas9) technology has revolutionized the gene therapy landscape.^[^ ^22^, ^23^ ^]^ In the context of cancer immunotherapy, CRISPR‐Cas9 has been extensively utilized for the screening of novel therapeutic targets,^[^ ^24^ ^]^ comprehending drug resistance mechanisms,^[^ ^25^ ^]^ and the development of new generations of chimeric antigen receptor (CAR)‐T cells.^[^ ^26^, ^27^ ^]^ However, these approaches are predominantly conducted in vitro or ex vivo and, when translated in vivo, are dependent on viral vectors. While these vectors exhibit efficient transfection capabilities, they carry the risk of genomic integration, off‐target modifications, and immunogenicity, potentially compromising immunological assessments and limiting the clinical translatability of this technology.^[^ ^28^, ^29^ ^]^ Addressing these challenges, several non‐viral vectors employing diverse materials (e.g., polymers, gold, lipids) have been explored to boost the in vivo delivery of CRISPR‐Cas9.^[^ ^30^, ^31^ ^]^

Lipid nanoparticles (LNPs) are emerging delivery tools for both RNA^[^ ^32^ ^]^ and DNA.^[^ ^33^ ^]^ LNPs are nanometric structures formed through the electrostatic complexation of a cationic lipidic phase with anionic nucleic acids dispersed in an aqueous phase.^[^ ^34^ ^]^ Following the successful use of LNPs to deliver small interfering RNA (siRNA),^[^ ^35^, ^36^ ^]^ messenger RNA (mRNA)^[^ ^36^ ^]^ and plasmid DNA (pDNA)^[^ ^37^ ^]^ for non‐viral immunomodulation of in vivo melanoma or colon cancer models, we have recently optimized LNPs for the encapsulation of Cas9 mRNA (mCas9) and sgRNA for in vitro and in vivo gene editing of green fluorescent protein (GFP) in GSCs.^[^ ^38^ ^]^

In the current study, we investigate the potential of using LNPs as a non‐viral CRISPR‐Cas9‐based screening tool for in vivo identification of immune therapeutic targets in GSCs. As a proof of concept, we chose to knock out (KO) two clinically important ICPs: CD47 and PD‐L1, which are overexpressed by GSCs. To test this, we employed a syngeneic GSCs model (NPE‐IE), that mirrors key features of the human mesenchymal GBMs.^[^ ^39^ ^]^ Here, we formulated LNPs encapsulating mCas9 and single guide RNA (sgRNA) against CD47 or PD‐L1 genes. We validated their gene editing ability in GSCs in vitro, followed by in vivo gene knock‐out assessment studies in orthotopic GSCs tumors following intratumoral administration. We evaluated the impact of knocking out of the proposed targets on tumor growth delay and TME phenotyping. Consistent with preclinical and clinical studies, we found that targeting PD‐L1 alone was not effective in reducing tumor burden, whereas CD47 treatment sensitized the tumor to the immune system and significantly reduced tumor growth. The proposed approach could be expanded for in vivo screening of novel ICPs specific to various GBM subtypes, ultimately identifying the most potent candidates for effective personalized GBM immunotherapy.

## 2 Results and Discussion

### 2.1 Formulation and Physicochemical Characterization of LNPs Encapsulating mCas9 and sgRNA

**Scheme 1:**
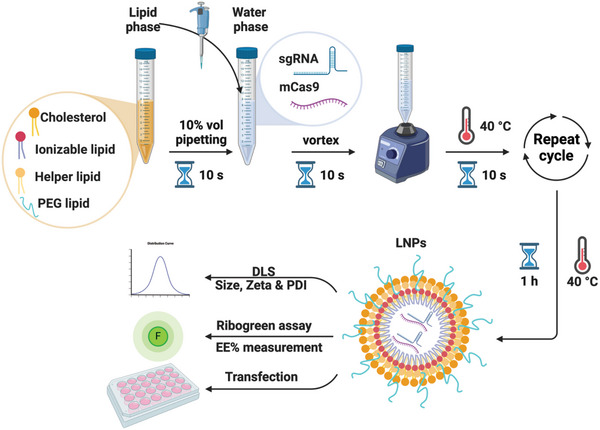
LNPs formulation method and characterization. Created with BioRender.com.

LNPs have become the preferred nanocarrier for the non‐viral delivery of nucleic acids, such as siRNA and mRNA, as demonstrated by the efficacy of Onpattro^[^ ^40^ ^]^ and COVID‐19 vaccines,^[^ ^41^, ^42^ ^]^ respectively. More recently, their clinical application has extended to include the delivery of mCas9/sgRNA for treating different genetic disorders (e.g., transthyretin amyloidosis^[^ ^43^ ^]^ hereditary angioedema,^[^ ^44^ ^]^ CEP290‐associated inherited retinal degeneration^[^ ^45^ ^]^). The current study proposes the application of CRISPR RNA LNPs in pre‐clinical settings as an in vivo screening tool for the identification of GSCs‐specific ICPs. To achieve this, ionizable LNPs were formulated to encapsulate mCas9 and sgRNA, utilizing a lipid composition that has been previously demonstrated to effectively incorporate siRNA,^[^ ^36^, ^46^ ^]^ mRNA,^[^ ^36^ ^]^ pDNA,^[^ ^46^ ^]^ and CRISPR RNAs^[^ ^38^ ^]^ (**Scheme** **1**). DLS measurements indicated that LNPs exhibited a particle size of approximately 150 nm, with no significant difference between LNPs targeting PD‐L1 (143 nm) or CD47 gene (152 nm) (**Table** **1**). The PDI of both formulations was < 0.150 (Table 1). As anticipated, due to the presence of the ionizable lipid DLin‐MC3‐DMA, at a physiological pH of 7.4, LNPs displayed a surface charge near neutral, with a Zeta potential of ≈5 mV (Table 1). Ribogreen assay suggested a high EE% (>83) of RNA in both formulations (Table 1). Since the Ribogreen assay cannot differentiate between various RNA types, it was necessary to further verify the successful encapsulation of both mCas9 and sgRNAs within the LNPs. For this purpose, unencapsulated RNA was initially digested with RNase A, followed by proteinase K treatment to neutralize RNase A activity. Subsequently, LNPs were disassembled using heparin to release the encapsulated RNA. The released RNA and their respective controls (mCas9, sgRNAs, and the mixture of mCas9/sgRNA) were analyzed using agarose gel electrophoresis (Figure S1, Supporting Information). Comparable intensities of mCas9/sgRNA ratios were measured for both the mCas9/sgRNA mixture (1.58) and those extracted from the LNPs (1.65) (Figure S1, Supporting Information). While this assay provides a semi‐qualitative analysis, it confirmed the encapsulation of both mCas9 and sgRNA within the LNPs.

**Table 1:** Physicochemical characterization and RNA encapsulation efficiency of LNPs formulation.

Target	Size [d.nm]^a^ ^),^ ^d^ ^)^	PDI^a^ ^),^ ^d^ ^)^	Charge [mV] ^b^ ^),^ ^d^ ^)^	mCas9/sgRNA EE [%]^c^ ^),^ ^d^ ^)^
PD‐L1	143.44 ± 5.06	0.095 ± 0.03	4.35 ± 0.76	83. 95 ± 3.96
CD47	152.10 ± 4.40	0.128 ± 0.04	5.83 ± 1.45	89.25 ± 3.54

^a)^ Measured by dynamic light scattering;

^b)^ Surface charge measured by electrophoresis;

^c)^ Percentage Encapsulation Efficiency (%EE) measured using Quant‐iT™ RiboGreen™ RNA Assay Kit; and

^d)^ Expressed as mean ± SD (n = 3).

### 2.2 sgRNAs Designed Against PD‐L1 and CD47 Are Functional and Specific

**Figure 1:**
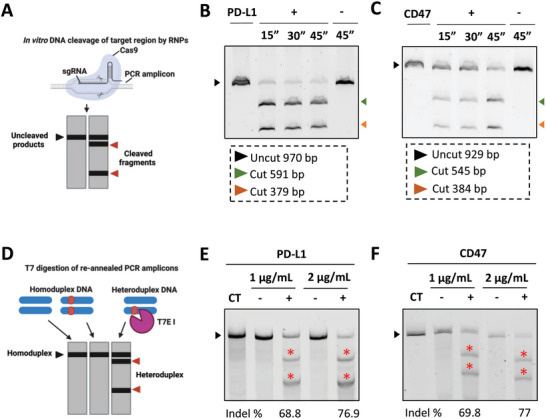
LNPs efficiently mediated in vitro PD‐L1 and CD47 gene editing in NPE‐IE cells. PD‐L1 and CD47 sgRNA specificity was validated using an in vitro Cas9 nuclease assay A). Briefly, PD‐L1 and CD47 amplicons were incubated with ribonucleic protein (RNP) complexes composed of commercial Cas9 nuclease a negative sgRNA (−) or targeting sgRNA against PD‐L1 or CD47 (+). Agarose gel electrophoresis of RNP digested PD‐L1 B) and CD47 C) amplicons. Black arrows indicate full‐length amplicons and coloured arrows represent the cleavage products. LNPs mediated in vitro PD‐L1 and CD47 gene editing was assessed using T7 mismatch assay D) as follows. Briefly, NPE‐IE cells were transfected with LNPs for 48 h at a concentration of 1 or 2 µg mL^−1^ of nucleic acids composed of mCas9 and a negative sgRNA (−) targeting sgRNA against PD‐L1 or CD47 (+). Gene knock‐out was analyzed by performing a T7 mismatch assay on DNA samples extracted 2‐ days post‐transfection D). Agarose gel electrophoresis of T7 digested PD‐L1 E) and CD47 F) amplicons. The percentage (%) of indel formation was calculated using the following formula = 100 × (∑ cleaved products/∑ cleaved products, full‐length amplicons). Schemes in A and D were created with BioRender.com.

PD‐L1 and CD47 have shown potential in preclinical studies as targets for GBM immunotherapy in murine models.^[^ ^13^, ^14^, ^47^, ^48^ ^]^ While clinical trials for PD‐L1 have yielded positive outcomes for a small subset of GBM patients, the majority have seen limited benefits.^[^ ^49^, ^50^ ^]^ There is a lack of investigation of the efficacy of these targets in mesenchymal GBM. In this study, PD‐L1 and CD47 were selected to validate the proof‐of‐concept of the proposed LNP‐based screening tool, given their established roles in immune modulation. Before assessing the LNPs' capability to induce in vitro gene knock‐out in cultured cells, the specificity of PD‐L1 and CD47 sgRNAs for their target sites was evaluated using an in vitro Cas9 nuclease assay (**Figure** **1**A). After incubating PCR amplicons with ribonucleic protein (RNP) comple consisting of Cas9 protein and sgRNA, the specificity of the sgRNA is determined by the appearance of new additional fragments on the agarose gel electrophoresis. This indicated that the designed sgRNA could effectively guide the Cas9 protein to the specific genomic site. Agarose gel images showed that incubating PD‐L1 (970 bp) and CD47 (929 bp) amplicons with target‐specific RNP (+) generated smaller fragments of ≈591/379 bp and 545/384 bp for PD‐L1 and CD47, respectively (Figure  1B,C). The emergence of these additional fragments was consistent with the anticipated cleavage products. For both PD‐L1 and CD47, successful DNA cleavage was detected after 15 min incubation. In both instances, DNA amplicons incubated with RNP comprising Cas9 and negative sgRNA (−) maintained the same size as the intact PD‐L1 and CD47 amplicons, and no additional cleavage products were observed.

### 2.3 LNPs Efficiently Mediated in vitro PD‐L1 and CD47 Gene Editing in NPE‐IE Cells

LNPs targeting PD‐L1 or CD47 were employed to transfect NPE‐IE GSCs or GL261 GBM cells for 48 h. While our primary focus was on NPE‐IE GSCs, we introduced GL261 cells for validation purposes, as they are the most commonly used murine GBM model in preclinical studies. The T7 mismatch assay was used to identify target‐specific gene editing events. Denaturation and re‐annealing of PCR amplicons carrying indels can lead to the formation of heteroduplexes. These mismatches are recognized by the T7 endonuclease (T7E I), which cleaves the DNA, resulting in the production of smaller fragments (Figure  1D). Upon denaturation and re‐annealing of PCR products derived from NPE‐IE cells, cells transfected with targeting LNPs (+) exhibited additional bands, signifying the presence of heteroduplexes between amplicons of varying lengths (e.g., wild type and edited amplicons) (Figure  1E,F). These additional bands indicate that an aberrant non‐homologous end joining (NHEJ) event occurred following LNPs‐mediated CRISPR‐Cas9 cleavage. Conversely, only a single band was evident in samples treated with negative LNPs (−), displaying a size comparable to untreated control (CT). The calculated indel percentages for PD‐L1 were ≈69% and 77% for cells treated with 1 and 2 µg mL^−1^, respectively. Similar percentages were obtained for CD47, exhibiting indel formation of 70% for the 1 µg mL^−1^ dose and 77% for the 2 µg mL^−1^ dose. While successful gene editing was confirmed for both targets in NPE‐IE cells, intriguingly, in GL261 cells, all re‐annealed samples matched untreated and negative controls (data not shown), implying the absence of any gene editing event in this cell line. Since the T7 endonuclease can only identify mismatches larger than 1 base, often underestimating the gene editing efficiency, further ICE analysis was performed on GL261 using trace data obtained from Sanger sequencing. Gene knock‐out was still not detectable (0%) for CD47 samples and was minimal (≈1%) for PD‐L1 samples (Figure S2A, Supporting Information). These significant differences in transfection efficiency between NPE‐IE and GL261 cells may be due to mechanistic factors affecting LNPs trafficking and endosomal escape between the two cell lines. In a screening study of 30 cell lines, Sayers et al. highlighted that transfection efficiency is influenced by several factors, including the organization of an endolysosomal system, endosomal pH, and lysosomal trafficking.^[^ ^51^ ^]^ Cell lines with low transfection efficiency often exhibit slower trafficking of LNPs from the endosome to the lysosome, along with defective endocytic membrane organization and acidification.

### 2.4 LNPs‐Mediated in vitro CD47 Gene Editing Resulted in Efficient CD47 Protein Knock‐Out in NPE‐IE Cells

**Figure 2:**
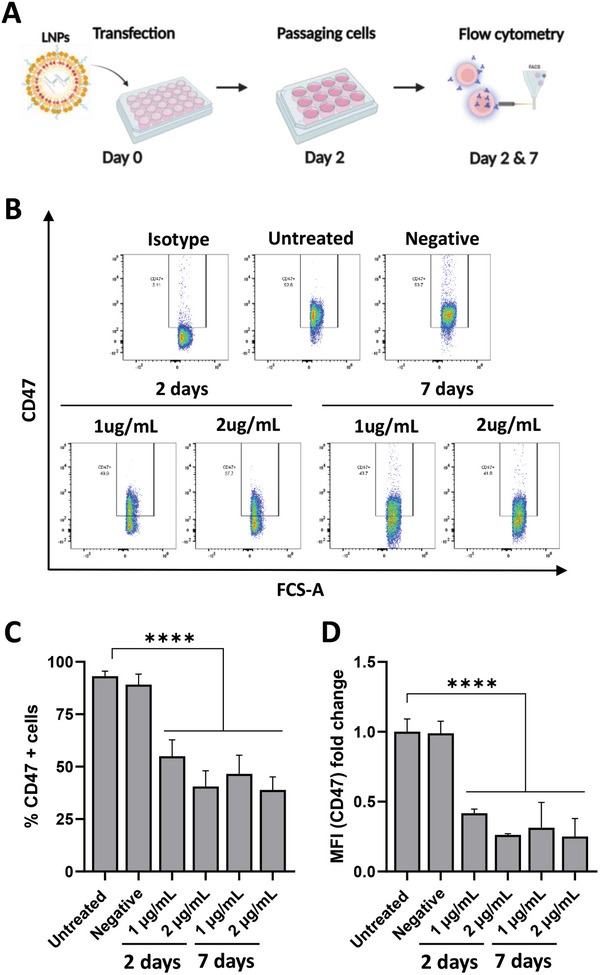
LNPs‐mediated in vitro CD47 gene editing resulted in efficient CD47 protein knock‐out in NPE‐IE cells. Schematic workflow of the experimental procedure A) briefly, NPE‐IE cells were transfected with LNPs for 48 h at a concentration of 1 or 2 µg mL^−1^ of total nucleic acids composed of mCas9 and a negative sgRNA (Negative) or CD47 targeting sgRNA. Protein knock‐out was measured at 2‐ and 7‐days post‐transfection using flow cytometry. Representative dot blots of untreated, isotype controls, cells transfected with negative LNPs, and cells transfected with CD47 targeting LNPs B) percentages (%) of CD47 positive cells (CD47^+^) C) median fluorescence intensity (MFI) of CD47 expression D) calculated on the entire cell population at 2‐ and 7‐days post‐transfection expressed as fold changed relative to untreated. Results are expressed as means ± SD, (*n* = 3). Significant differences are presented as ^****^ *p* < 0.0001 compared to untreated control using one‐way ANOVA. The scheme in A was created with BioRender.com.

Following successful in vitro PD‐L1 and CD47 gene editing confirmation in NPE‐IE cells, the impact of gene editing on protein expression was assessed. As surface PD‐L1 protein expression was absent in in vitro cultured NPE‐IE cells, though not the case in vivo, this analysis was solely conducted for CD47. To this end, NPE‐IE cells were transfected with LNPs_sgCD47_ for 48 h (**Figure**  **2**A). Cells were subsequently expanded for an additional 5 days (Figure  2A). CD47 expression was evaluated via flow cytometry on days 2 and 7 post‐transfection (Figure  2B). Transfection with LNPs_sgCD47_ led to a significant reduction in CD47 levels (*p* < 0.0001) (Figure  2C,D). The CD47‐positive (CD47^+^) cells (%) values for untreated controls were ≈93%. Two days post‐transfection CD47^+^ cells significantly dropped to ≈55% and ≈40% for RNA doses of 1 and 2 µg mL^−1^, respectively (Figure  2C). CD47 protein knock‐out efficiency peaked at ≈54% on day 7 post‐transfection for 2 µg mL^−1^ concentration (Figure  2C). Cells transfected with negative LNPs were comparable to untreated controls (Figure  2C,D). Conversely, flow cytometry analysis of GL261 cells transfected with LNPs_sgPD‐L1_ or LNPs_sgCD47_ showed no alterations in PD‐L1 (Figure S2B,C, Supporting Information) or CD47 protein expression (Figure S2D,E, Supporting Information). These findings aligned with the T7 mismatch assay and ICE data, demonstrating the lack of gene editing in GL261 cells and confirming the successful editing of NPE‐IE cells using LNPs.

### 2.5 Orthotopic NPE‐IE Tumors Presented Higher Levels of Immune Cell Infiltration Compared to GL261 Tumors

**Figure 3:**
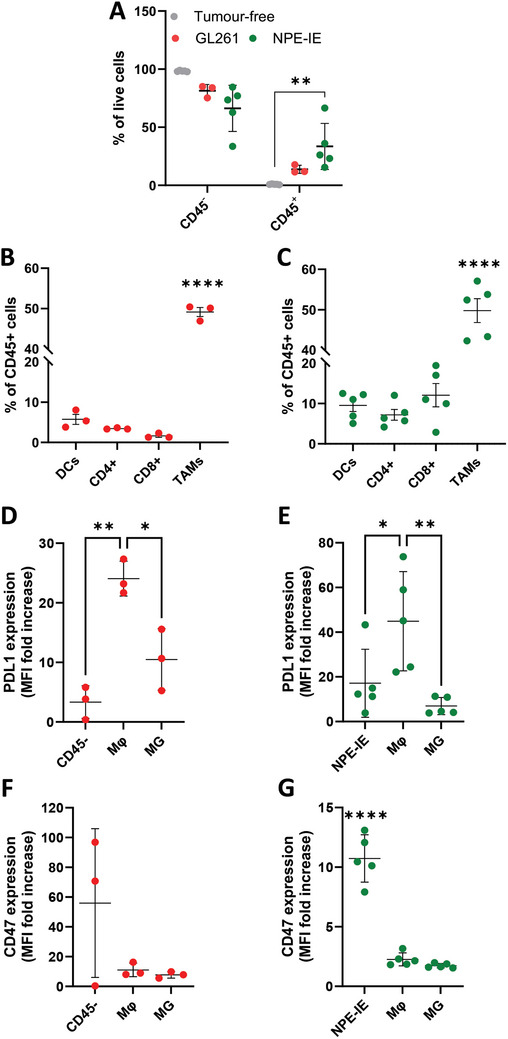
Enhanced immune cell infiltration in orthotopic NPE‐IE tumors compared to GL261 orthotopic tumors. GL261 (*n* = 3) or NPE‐IE (*n* = 5) tumors extracted from perfused C57BL/6 mice brains were dissected and enzymatically digested to obtain a single‐cell suspension for phenotyping by flow cytometry. Tumor‐free brain tissues (*n* = 5) were used as negative controls. Quantification of the incidence of CD45‐negative (CD45^−^) and CD45‐positive (CD45^+^) cells in tumor‐free and GL261 tumor‐bearing brains A). Quantification of different immune cell (CD45^+^) populations in the tumor microenvironment of GL261 B) and NPE‐IE C) tumors, identified as follows: dendritic cells (DCs) (CD45^+^/CD11b^+^/F4/80^−^/CD11c^+^), CD4^+^ cells (CD45^+^/CD4^+^), CD8^+^ cells (CD45^+^/CD8^+^), and tumor‐associated macrophages/microglia (TAMs) (CD45^+^/CD11b^+^/F4/80^+^). Median fluorescence intensity (MFI) of PD‐L1 D,E) and CD47 F,G) for GL261 D,F) and NPE‐IE E,G) tumors, calculated on the whole population of CD45^−^ cells (GL261), tumor cells (NPE‐IE), macrophages (Mϕ) (CD45^high^/CD11b^+^/F4/80^+^), and microglia (MG) (CD45^med^/CD11b^+^/F4/80^+^). The MFI of each population was normalized to the corresponding fluorescence minus one (FMO) signal. Results are expressed as means ± SD. Each point represents an individual mouse. Significant differences were presented as ^*^ *p* < 0.05, ^**^ *p* < 0.01, ^***^ *p* < 0.001, ^****^ *p* < 0.0001 using one‐way ANOVA.

Prior to validating the LNPs in vivo, multiparametric flow cytometry analysis was conducted to characterize the TME of GL261 and NPE‐IE orthotopic tumors. GL261 and NPE‐IE tumors exhibited increased infiltration of immune cells (CD45^+^) compared to healthy brain tissue (**Figure**  **3**A). Immune cell infiltration constituted 33.5 ± 17%, 13.7 ± 3%, and 0.9 ± 0.3% of live cells in NPE‐IE, GL261, and tumor‐free brain parenchymal tissue, respectively, in the following order NPE‐IE > GL261 > tumor‐free brain parenchymal tissue. As anticipated, TAMs (CD45^+^/CD11b^+^/F4/80^+^) constituted a significant portion (≈49%) of infiltrating immune cells in both GL261 (Figure  3B) and NPE‐IE (Figure  3C) tumors. In contrast, all other cell populations analyzed comprised, on average, ≤15% of the total leukocyte population (GL261 Figure  3B and NPE‐IE Figure  3C).

### 2.6 Both Tumor Models Express CD47 and PD‐L1 in vivo

Next, we aimed to assess the baseline levels of both ICPs in the two tumor models. In GL261 tumors, the mean fluorescence intensity(MFI) of PD‐L1 signal in macrophages (CD45^high^/CD11b^+^/F4/80^+^) was approximately twofold higher than in microglia (CD45^med^/CD11b^+^/F4/80^+^) (*p* < 0.05) and approximately sevenfold higher than in CD45^−^ cells (*p* < 0.01) (macrophages > microglia > CD45^−^) (Figure  3D). In NPE‐IE tumors, the MFI of PD‐L1 signal in macrophages was ≈2.5‐fold higher than in tumor cells (*p* < 0.05) and ≈6.5‐fold higher than in microglia (*p* < 0.01) (Figure  3E) (macrophages > tumor cells > microglia). For CD47, the highest CD47 expression was observed in tumor cells in GL261 and NPE‐IE tumor cells (Figure  3F,G) while macrophages and microglia displayed minimal CD47 expression (Figure  3F,G). Overall, CD47 expression was more pronounced in GL261 tumors, while PD‐L1 expression was higher in NPE‐IE tumors. Both CD47 and PDL1 have been extensively reported to be expressed in patient GBM tissues, as well as murine and human cell lines, with expression levels varying depending on tumor subtype and progression stage.^[^ ^47^, ^52^ ^]^ Clinical data have shown that tumor samples from the mesenchymal GBM subtype have the highest PD‐L1 expression compared to the other subgroups,^[^ ^53^ ^]^ which is consistent with our observation of higher PD‐L1 expression in NPE‐IE tumors compared to GL261.

### 2.7 LNPs Are Taken up Efficiently by Cancer and Immune Cells in vivo

**Figure 4:**
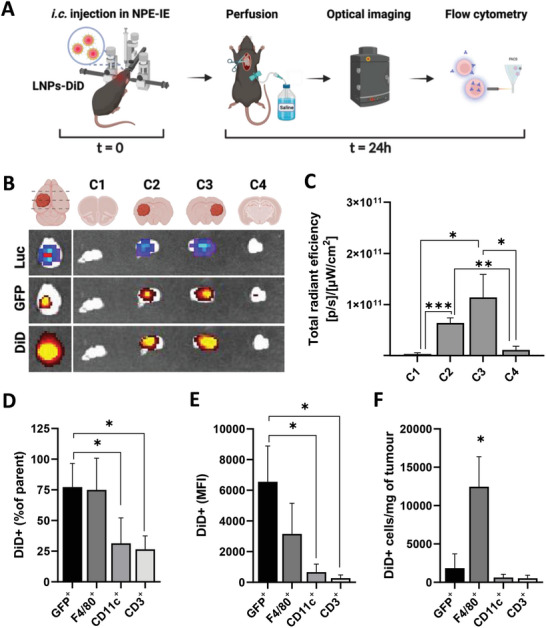
LNPs administered intracranially in orthotopic NPE‐IE tumors can be distributed to both cancer and immune cells. Schematic representation of experimental procedure A). Briefly, C57BL/6 mice bearing orthotopic NPE‐IE tumors (*n* = 3) were intracranially (i.c.) injected with LNPs labeled with NIR dye (DiD). Twenty‐four hours post‐injection animals were sacrificed and transcardially perfused with saline. One mouse was left untreated and used as a negative control. Brain tissues were imaged using the IVIS Lumina III system. Tumors extracted from perfused mouse brains were dissected and enzymatically digested to obtain a single‐cell suspension, which was assessed for viability and stained for flow cytometry analysis. Representative optical images of brains and brain sections. Bioluminescence and fluorescence expression of Luciferase (Luc) and Green Fluorescence Protein (GFP) indicate the presence of the tumor, while DiD signal is representative of the LNPs distribution B). Semi‐quantitative analysis of the DiD signal present in the coronal sections C). Flow cytometry quantification of LNPs_DiD_ uptake within the tumor microenvironment (TME) D–F). Percentages of DiD+ cells among cancer cells (GFP^+^) and various immune cell sub‐populations (F4/80^+^/CD11c^+^/CD3^+^) expressed as percentage of their respective parent population (D). Median fluorescence intensity (MFI) of DiD signal in each cell population (E). Absolute counts of DiD+ cells for each cell population (F). Absolute counts were obtained by including precision counting beads prior to acquisition on the flow cytometer. Results are expressed as mean ± SD. Significant differences are presented as ^*^ *p* < 0.05, ^**^ *p* < 0.01, ^***^ *p* < 0.001 using an unpaired *t*‐test. The scheme in A was created with BioRender.com.

To examine the cellular distribution of LNPs within the TME, DiD‐labeled LNPs were intracranially administered in NPE‐IE orthotopic tumors (**Figure**  **4**A). Locally administered LNPs were retained within the tumorous tissues (Luc+/GFP+), as confirmed by the co‐localization of Luc, GFP, and DiD signals, further supported by the highest signals observed in coronal sections containing tumorous tissues (Figure  4B,C). The order of % DiD+ cells was cancer cells (GFP^+^) ≈77% > TAMs (F4/80^+^) ≈75% > dendritic cells (CD11c^+^) ≈31% > T‐cells (CD3^+^) ≈26% (Figure  4D), this order was reflected in the respective MFI values (Figure  4E). F4/80^+^ incidence in the TME was significantly (*p* < 0.05) higher than the other cells, making TAMs the primary contributor to LNPs uptake within the TME, followed by cancer cells (GFP^+^) (Figure  4F). The enhanced affinity of LNPs for TAMs may be attributed to the phagocytic capabilities of the TAMs. However, it remains to be determined whether this also correlates with efficient transfection. Given the critical role of TAMs in promoting immunosuppression in GBM, future studies should explore the potential of using LNPs to edit TAMs, aiming to activate them to attack cancer cells.

### 2.8 Intracranial Injection of LNPs Effectively Facilitates PD‐L1 and CD47 Gene Editing in GL261 and NPE‐IE Orthotopic Tumors

**Figure 5:**
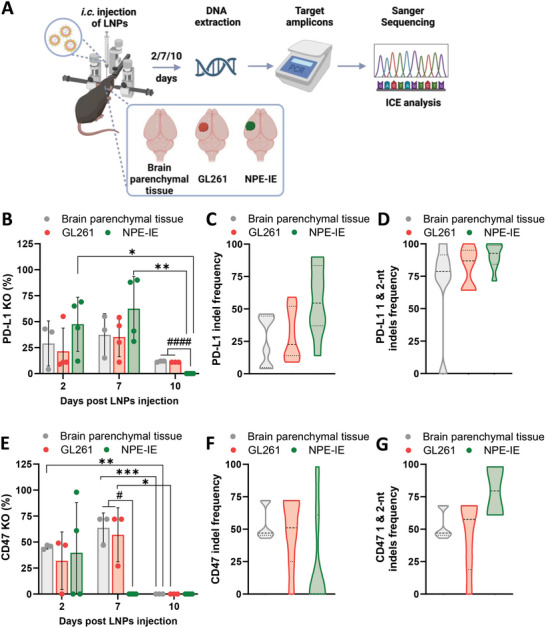
Intracranial injection of LNPs effectively facilitates PD‐L1 and CD47 gene editing in tumor‐free brain parenchymal tissue as well as GL261 and NPE‐IE orthotopic tumors. Schematic representation of experimental procedure A). Briefly, LNPs encapsulating mCas9 and targeting sgRNA against PD‐L1 (LNPs_sgPD‐L1_) or CD47 (LNPs _sgCD47_) were intracranially (i.c.) injected into tumor‐free brain parenchymal tissue (*n* = 3) or GL261/NPE‐IE tumors (*n* = 3–4), at a nucleic acid dose of 15 µg. Animals were sacrificed at 2, 7, and 10 days post‐injection, brain/tumorous tissues were harvested, and DNA was extracted for PCR amplification, Sanger Sequencing, and Interference of CRISPR Edits (ICE) analysis. Percentages of PD‐L1 B) and CD47 E) gene knock‐out (KO). Violin plots showing the combined (day 2 and 7) distribution of indel frequency for PD‐L1 C) and CD47 F). Violin plots showing the distribution of the incidence of 1‐ and 2‐ nucleotides (nt) indels following PD‐L1 D) and CD47 G) gene editing. Results in B and E are expressed as means ± SD. Each point represents an individual mouse. The dark dotted line in the middle of the violin plots C, D, F, and G represents the median, the top dotted line represents the 3rd quartile, and the bottom dotted line indicates the 1st quartile of the data set. Significant differences are presented as ^*^ *p* < 0.05, ^**^ *p* < 0.01, ^***^ *p* < 0.001 within each experimental group and as ^#^ *p* < 0.05, ^####^ *p* < 0.0001 for each time point, respectively, using one‐way ANOVA. The scheme in A was created with BioRender.com.

In vivo, gene editing of PD‐L1 or CD47 was investigated through intracranial administration of LNPs targeting PD‐L1 (LNPs_sgPD‐L1_) or CD47 (LNPs_sgCD47_) in both tumor‐free brain parenchymal tissue, as control tissues, and brains hosting orthotopic GL261 or NPE‐IE tumors (**Figure**  **5**A). GL261 tumors were included in the in vivo study despite negative in vitro knock‐out data as our previous studies and those of other groups have demonstrated that in vitro and in vivo transfection do not always correlate^37^, ^54^, ^55^ (37, 54, 55).

ICE analysis of Sanger sequencing DNA traces revealed average PD‐L1 knock‐out efficiency in tumor‐free brain parenchymal tissue (29%), GL261 (21%), and NPE‐IE (47%) tumors, at 2 days post LNPs injection (Figure  5B). By day 7, gene knock‐out increased by ≈10% across all three conditions. Surprisingly, by day 10, PD‐L1 knock‐out significantly decreased to ≈11% in tumor‐free brain parenchymal tissue and GL261 tumors and was undetectable in NPE‐IE tumors (0%). On the other hand, CD47 gene KO% was confirmed in tumor‐free brain parenchymal tissue (45%), GL261 (32%), and NPE‐IE (40%) tumors (Figure  5E). Although tumor‐free brain parenchymal tissue (64%) and GL261 models (61%) maintained levels of gene editing until day 7, CD47 KO was undetectable in NPE‐IE tumors (0%). By day 10, CD47 gene knock‐out levels had converged to 0% for all conditions. The combined indel profile pattern for day 2 and 7 of PD‐L1 (Figure S4A, Supporting Information) and CD47 (Figure S4B, Supporting Information) was further examined, showing apparent median indel frequency values of 29%, 22%, and 54% (PD‐L1) and 47%, 51%, and 0% (CD47) for tumor‐free brain parenchymal tissue, GL261, and NPE‐IE tumors, respectively (Figure  5C,F). The dominant indels for both targets involved frameshift insertions or deletions of 1 or 2 nucleotides (nt) from the target site (Figure  5D,G) with occurrence being more prominent in NPE‐IE cells.

Our results at day 2 indicate that LNPs effectively edited both tumor models and brain parenchymal tissue. However, there was a substantial reduction in PD‐L1 and CD47 gene knock‐out (CD47 > PD‐L1) by days 7 and 10 compared to day 2. Since gene knock‐out is a permanent event, it was hypothesized that the edited cells contributing to gene knock‐out scores at the early time points may have been eliminated by the immune system. While this has yet to be demonstrated, it may explain why gene knock‐out scores converged to 0 by day 7 (CD47) and day 10 (PD‐L1 and CD47). This effect was most pronounced in NPE‐IE tumors, followed by the GL261 model and tumor‐free brain parenchymal tissue, likely due to the higher immune cell infiltration observed in NPE‐IE tumors. Additionally, our results suggested faster clearance of CD47‐edited cells compared to PD‐L1, more prominently in NPE‐IE tumors. PD‐L1 knock‐out activates T cells via multiple pathways culminating in the expansion of helper CD4^+^ and cytotoxic CD8^+^ T cells to induce an anti‐tumor response.^[^ ^9^ ^]^ Conversely, CD47 gene knock‐out prompts TAMs to recognize and phagocytose cancer cells, thus facilitating antigen presentation to T cells.^[^ ^56^ ^]^ While TAM‐mediated phagocytosis is relatively prompt, a T cell‐mediated immune response requires a more protracted timeline for an effective adaptive response to occur. Differences in the timeline required to induce a response and abundance of TAMs versus T cells could explain the faster clearance of CD47 knock‐out cells compared to PD‐L1. Other studies that reported successful non‐viral PD‐L1^[^ ^57^ ^]^ and CD47^[^ ^58^ ^]^ gene editing in B16F10‐OVA tumors examined tissues within a short timeframe (≤2 days) following the last injection. A direct comparison with our study could therefore not be made to conclude on the clearance pattern. In a different context, one study showed that AAV‐based gene editing of low‐density lipoprotein receptor (LDLR) led to indels forming within the first 6 weeks post‐injection (5 to 20%), which then vanished at 12 weeks.^[^ ^59^ ^]^ The clearance of edited hepatocytes was linked to a robust memory T cell response in SaCas9‐immunised mice, but not in OVA‐immunised mice. In our investigation, where immunization was not involved, the clearance of edited cells may suggest their vulnerability to the immune system post‐PD‐L1 and CD47 editing.

It was interesting to observe that despite unsuccessful in vitro knock‐out studies for the GL261 model, successful gene editing was observed in vivo. One plausible explanation is the presence of other cell types in the TME (e.g., immune and endothelial cells, cancer‐associated fibroblasts etc.^[^ ^60^ ^]^) compared to the monoculture used in vitro. Additionally, our biodistribution studies also showed that LNPs have a high affinity for cancer cells and TAMs following intratumoral administration. Therefore, ICE analysis for in vivo samples could represent knock‐out efficiency across multiple cell types. Another hypothesis is that the TME alters ICPs expression profiles on cancer cells and their affinity to LNPs compared to in vitro settings, favoring successful gene knock‐out compared to in vitro.

Given the faster clearance rates of edited cells observed in NPE‐IE tumors and the higher infiltration of immune cells compared to GL261, we hypothesized that NPE‐IE tumors could provide a more suitable model for assessing the impact of gene knock‐out on the immune response and tumor growth delay.

### 2.9 LNPs Targeting Both PD‐L1 and CD47 Enhance Immune Cell Infiltration in Orthotopic NPE‐IE Tumors

**Figure 6:**
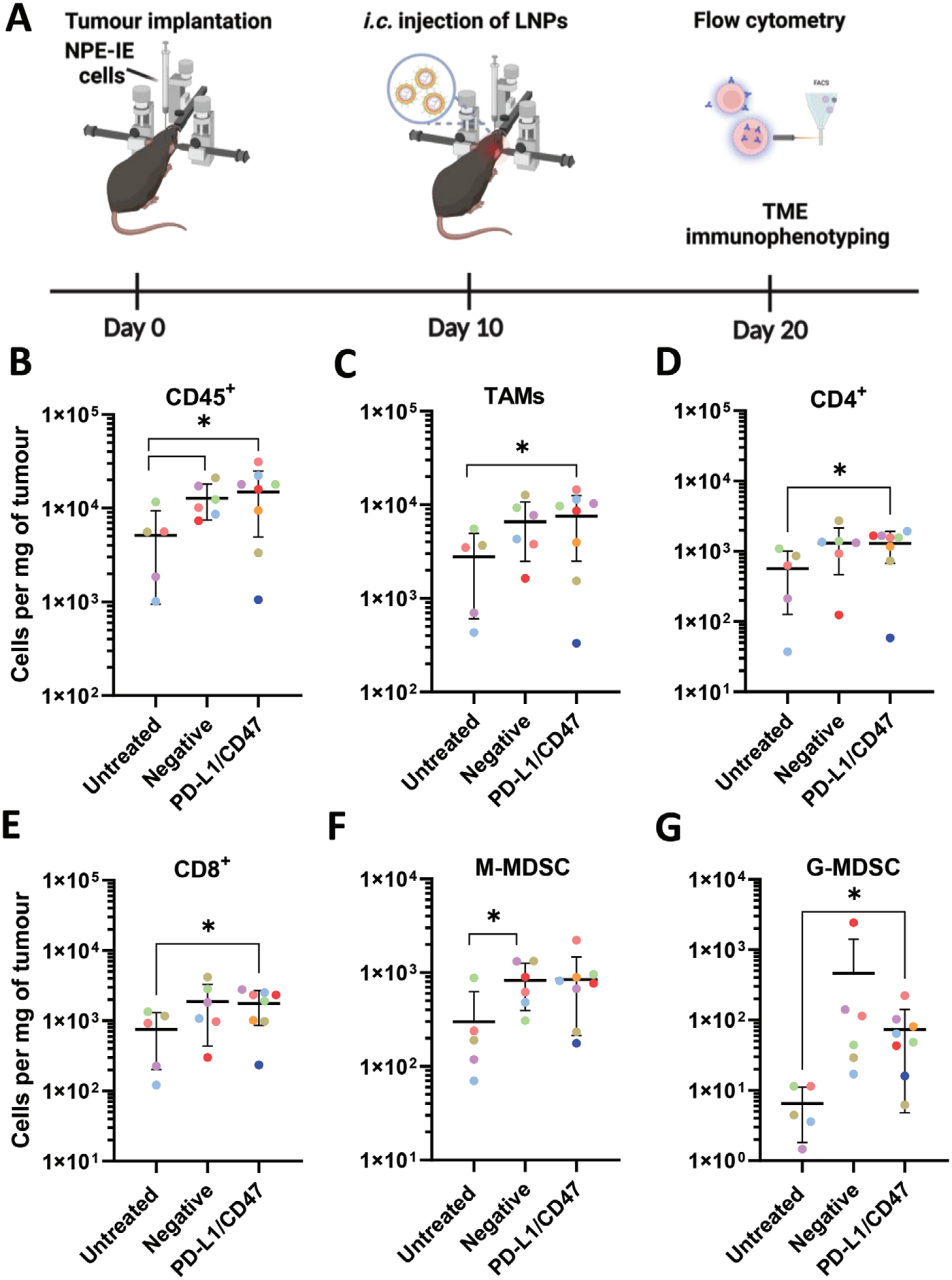
LNPs targeting both PD‐L1 and CD47 enhance immune cell infiltration in orthotopic NPE‐IE tumors. Schematic representation of experimental procedure A). Briefly, C57BL/6 mice bearing orthotopic NPE‐IE tumors were intracranially (i.c.) injected with LNPs encapsulating mCas9 and a combination of targeting sgRNA against PD‐L1 and CD47 (PD‐L1/CD47, *n* = 8), or non‐targeting sgRNA (negative, *n* = 6), at a nucleic acid dose of 15 µg. One group was left untreated (*n* = 5). Ten days post‐injection animals were sacrificed and tumors extracted from perfused mouse brains were dissected and enzymatically digested to obtain a single‐cell suspension, which was assessed for viability and stained with anti‐mouse CD45, CD11b, Ly6G, F4/80, CD11c, CD4, CD8. Absolute counts of total immune cells (CD45^+^) B), tumor‐associated macrophages/microglia (TAMs) (C), CD4^+^cells (D) and CD8^+^cells (E), monocytic‐myeloid derived suppressor cells (M‐MDSC) (F), and granulocytic‐myeloid derived suppressor cells (G‐MDSC) (G) expressed as cells per mg of tumor. Absolute counts were obtained by including precision counting beads prior to acquisition on the flow cytometer. Results are expressed as means ± SD. Each point represents an individual mouse. Significant differences are presented as ^*^ *p* < 0.05 using an unpaired *t*‐test. The scheme in A was created with BioRender.com.

To investigate the immunomodulatory effects following dual targeting of PD‐L1 and CD47, a single intracranial administration of LNPs encapsulating a total of 15 µg of RNA was performed in NPE‐IE tumors (**Figure**  **6**A). Control groups included untreated animals and those injected with non‐targeting RNA. Interestingly, a significant increase (p < 0.05) in total immune cell count was observed in both the negative and PD‐L1/CD47 groups (Figure  6B), suggesting that LNPs are not inert and could enhance immune cell infiltration in the tumor. This was consistent with our previous studies in B16F10 tumors following intratumoral administration of LNPs encapsulating siRNA, mRNA or pDNA.^[^ ^35^, ^61^ ^]^ It also aligns with literature reports on the intrinsic adjuvant properties of ionizable LNPs, observed with both empty LNPs^[^ ^62^ ^]^ and mRNA‐LNPs^[^ ^42^, ^62^, ^63^, ^64^ ^]^ administered through various routes.

TAMs, CD4^+^, CD8^+^ cells were significantly enriched (p < 0.05) in the PD‐L1/CD47 group but not in the negative control (Figure  6C–G), suggesting a potentiated anti‐tumor response. The levels of dendritic cells (DCs) and the CD4^+^/CD8^+^ ratio remained stable between LNPs‐treated animals and untreated controls (Figure S7B,C, Supporting Information). Both negative and positive LNPs‐treated groups exhibited increased levels of monocytic (M‐MDSCs) and granulocytic (G‐MDSCs) myeloid‐derived suppressor cells (MDSCs), respectively, compared to untreated controls. M‐MDSC cells were significantly (*p* < 0.05) more abundant in the negative group (Figure  6F), while G‐MDSCs were significantly enriched (*p* < 0.05) in the PD‐L1/CD47 group. The presence of MDSCs is a recurring observation in mouse and human GBM as well as other tumors^[^ ^65^, ^66^ ^]^ which were found to correlate with poor prognosis and resistance to various ICPs therapies. The increased MDSCs levels could be an adaptive counter‐response to enhanced immune cell infiltration and specifically T cells to the TME. Notably, an initial MDSCs increase following ICPs therapy has been observed in melanoma patients.^[^ ^67^ ^]^

### 2.10 LNPs‐Mediated CD47 Targeting Resulted in Reduced Tumor Growth

**Figure 7:**
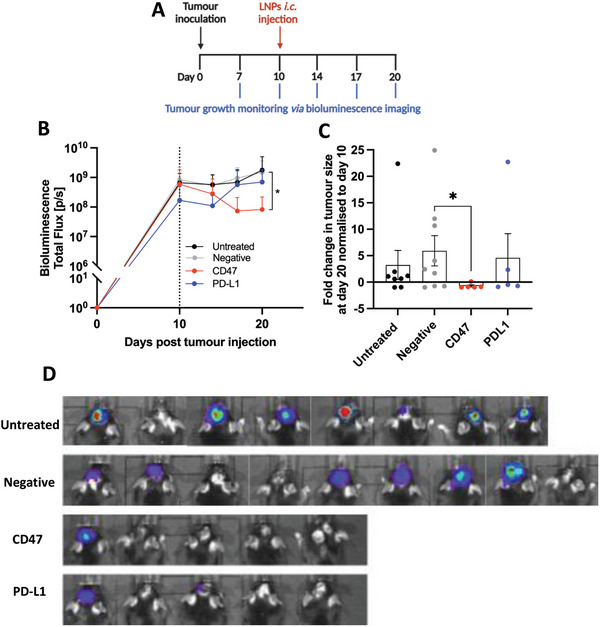
LNPs‐mediated CD47 targeting resulted in reduced tumor growth. Schematic representation of the experimental procedure A). Briefly, C57BL/6 mice bearing orthotopic NPE‐IE tumors were intracranially (i.c.) injected with LNPs encapsulating mCas9 and targeting sgRNA against PD‐L1 (PD‐L1, *n*  =  5), or CD47 (CD47, *n*  =  5) or non‐targeting sgRNA (Negative, *n*  =  9), at a nucleic acid dose of 15 µg. One group was left untreated (*n*  =  8). Bioluminescence Intensity was measured using the IVIS Lumina III system to assess changes in the tumor growth post‐LNP treatment. Tumor growth curves are expressed as the total flux of the BLI (photons/seconds) over time B). Bar chart representative of the fold change in tumor size measured at the study endpoint (day 20), normalized to the size prior to LNPs treatment (day 10) C). Representative BLI images of mice at day 20 post tumor inoculation D); all images are reported at the same color scale, with min/max radiance of 10^7^/10^9^ photons/sec/cm^2^/sr. Results in tumor growth and fold change are expressed as means ± SD and means ± SEM, respectively. Significant differences are presented as ^*^ *p* < 0.05 using an unpaired *t*‐test, with a Mann–Whitney post‐test. The scheme in A was created with BioRender.com.

To understand the potential effect of targeting CD47 and PD‐L1 in sensitizing GSCs tumors, therapy studies were performed using LNPs targeting PD‐L1 (LNPs_sgPD‐L1_) or CD47 (LNPs_sgCD47_) following a single intracranial injection on day 10 post‐NPE‐IE tumor inoculation (**Figure**  **7**A). Negative controls comprised LNPs encapsulating non‐targeting sgRNA (LNPs_sgNeg_). LNPs administration did not cause any significant effect on body weight loss in all treatment conditions (Figure S8, Supporting Information). Treatment of mice with LNPs_sgCD47_ significantly inhibited tumor growth compared to LNPs_sgNeg_ (*p* < 0.05) group (Figure  7B). While all groups demonstrated an increase in tumor size from pre‐LNP treatment (day 10) to the endpoint (day 20), the CD47 group exhibited significantly reduced tumor size (Figure  7C,D; Figure S9, Supporting Information). Average tumor size on day 20 was negative (9.5 E^08^) > untreated (6.8 E^08^) > PD‐L1 (5.7 E^08^) > CD47 (7.4 E^07^) (Figure  7D). In this study, only CD47 treatment led to a significant delay in NPE‐IE tumor growth. This can possibly be explained by the high prevalence of TAMs (≈50% of CD45^+^ cells) in the TME.^[^ ^5^ ^]^ TAMs have the potential to initiate a fast and robust anti‐tumor immune response by directly phagocytosing CD47‐ knock‐out NPE‐IE cells and facilitating antigen presentation to T cells. On the other hand, the limited infiltration of T cells in the TME may explain the lower efficacy of PD‐L1 compared to CD47 targeting.^[^ ^68^ ^]^ This lack of efficacy in the PD‐L1 group aligns with clinical observations in GBM patients, where immunotherapies targeting the PD‐1/PD‐L1 axis have yielded limited success. The prognosis is particularly poor for patients who have exhibited a transition to a mesenchymal tumor state, associated with increased resistance to immunotherapy and shorter survival.^[^ ^69^ ^]^ Our knockout data in Figure  5 demonstrate efficient knockout of both CD47 and PD‐L1 and subsequent clearance of the edited cells. Despite this only CD47 knockout significantly delayed tumor growth, though it was insufficient for complete tumor eradication, which is anticipated. These tumors are highly aggressive and deploy multiple resistant mechanisms, thus, while edited subclones may be cleared, unedited GSCs continue to proliferate. Therefore, future studies will require a multifaced approach to achieve full tumor eradication. Effective therapy will likely require combining immunotherapy, which may include targeting multiple ICPs, potentially targeting multiple ICPs, with chemotherapy and radiotherapy to better replicate clinical conditions and enhance treatment effects. Future studies should involve larger cohorts of animals as well as long‐term survival assessments to evaluate the benefits of combination therapies over time. We envision the use of LNPs for the screening of a pool of ICPs, testing them alone or in combination with standard of care, as well as in combination with other ICPs, to ultimately determine the most potent therapeutic approach.

## 3 Conclusion

This study validates the use of LNPs as a proof‐of‐concept tool for expediting the in vivo screening of ICPs for GSCs/GBM immunotherapy. Intracranially administered LNPs loaded with mCas9/sgRNA effectively edited CD47 and PD‐L1 ICPs genes in the brain parenchyma and orthotopic GBM/GSCs models. CD47 emerged as a more promising target for mesenchymal GSCs, as compared to PD‐L1, highlighting its potential for GBM/GSCs immunotherapy. Integrating CD47 targeting with other treatment modalities such as chemo and radiotherapy, or additional ICPs, may further enhance its effectiveness. Larger treatment cohorts and future long‐term dose‐response studies are necessary to optimize this system. Ultimately, this approach aims to be applied in patient‐derived xenografts models to accelerate the identification translatability of personalized ICP blockade therapies tailored to specific GBM subgroups and patients.

## 4 Experimental Section

### Materials

N‐palmitoyl‐sphingosine‐1‐[succinyl(methoxy polyethylene glycol) 2000] (C16‐PEG2000), and 1, 2 dioleoyl‐sn‐glycero‐3‐phosphoethanolamine (DOPE) lipids were from Avanti Polar Lipids (USA). Dilinoleylmethyl‐4‐dimethylaminobutyrate (DLin‐MC3‐DMA) was from Biorbyt (UK). Cholesterol (CHOL), Triton X‐100, Amicon ultra 0.5 mL filters MW CO 30 kDa, and N‐(2‐Hydroxyethyl) piperazine‐N′‐(2‐ethanesulfonic acid) (HEPES) were from Sigma‐Aldrich (USA). Lipophilic dye DiIC18(5); 1,1′‐dioctadecyl‐3,3,3′,3′‐ tetramethylindodicarbocyanine, 4‐chlorobenzenesulfonate salt (DiD) was from Insight Biotechnology Limited (UK). CleanCap Cas9 mRNA – 5moU (mCas9) was from TriLink Biotechnologies (USA). Single guide RNAs (sgRNAs) targeting PD‐L1 and CD47 were designed using the Chop Chop online tool (https://chopchop.cbu.uib.no/) and purchased from Synthego (USA). MasterPure complete DNA & RNA purification kit was from Epicentre (USA). UltraPure agarose and TRIzol reagent were from Invitrogen (UK). 10 000x GelRed was from Biotium (UK). EnGen spy Cas9 NLS, Monarch PCR and DNA cleanup kit, OneTaq Hot Start 2X Master Mix, Gel Loading Dye Purple No SDS, and Proteinase K were from New England Biolabs (UK). MEM non‐essential amino acids (MEM NEAA), penicillin/streptomycin (P/S), bovine albumin fraction V 7.5% solution (BSA), 2‐mercaptoethanol (50 mm), B‐27 supplement (50X), N‐2 supplement (100x), phosphate‐buffered saline (PBS), GlutaMAX, fetal calf serum (FCS), 0.05% trypsin‐EDTA, 16% formaldehyde solution and Advanced RPMI 1640 media were from Thermo Fisher Scientific (UK). Glucose, accutase, and DMEM/HAMS F12 were from Sigma‐Aldrich (USA). Cultrex mouse laminin I was from Bio‐Techne (USA). Recombinant murine EGF and recombinant human FGF were from PeproTech (UK). All fluorophore‐conjugated anti‐mouse antibodies used in this study, Pacific Blue CD45 (#103 126), PerCP/Cyanine 5.5 CD11b (#101 228), PE F4/80 (#123 110), Brilliant Violet 650 CD11c (#117 339), FITC Ly‐6G (#127 605), Brilliant Violet 785 CD4 (#100 552), PE CD8 (#100 707), APC CD47 (#127 514), PE CD47 (#127 507), PE PD‐L1 (#124 308), PerCP/Cyanine5.5 CD3 (#100 218) were from Biolegend (USA) as well as Zombie Aqua and precision count beads. VetBond tissue adhesive was from Thermo Fisher Scientific (UK). D‐luciferin Potassium Salt was from Syd Labs (USA). Iso‐Vet was from Chanelle Pharma (IE). Euthatal was from Centaur Services (UK). Mersilk non‐absorbable sutures were from Aston Pharma (UK).

### Methods—Preparation of Lipid Nanoparticles (LNPs)

LNPs encapsulating mCas9 and sgRNA were prepared using the ethanol injection method with slight modifications (Scheme  1).^[^ ^36^ ^]^ For the preparation of the organic phase (Table S1, Supporting Information), cholesterol:Dlin‐MC3‐DMA:DOPE:C16‐PEG2000 (46.5:35:16:2.5 molar ratio) were dissolved in absolute ethanol, to which 6.19 µL of 20 mm citrate buffer (pH 4, in RNase‐free water) were added and heated at 60 °C for 5 min (min). Simultaneously, the aqueous phase (Table S1, Supporting Information) containing mCas9 and sgRNA (5 µg total nucleic acids) was dissolved in 100 µL of 20 mm citrate buffer (pH 4, in RNase free water) and incubated at 40 °C for 5 min. Next, 10 µL of the lipid phase was gradually added to the aqueous phase and mixed by pipetting for 10 s, followed by 10 s of the strong vortex, and 20 s of incubation at 40 °C. This procedure was repeated until the organic phase was fully consumed. LNPs were incubated at 40 °C for 1 h. Nitrogen (N_2_) was used to evaporate any residual ethanol in the sample. For in vitro studies, LNPs were concentrated to 25% of the original volume using Amicon ultra centrifugal filters with a 30 kDa cut‐off. For in vivo studies, concentrated LNPs were resuspended in 20 mm HEPES buffer (pH≈7). To prepare fluorescently labeled LNPs for in vivo tracking, lipophilic dye (DiD) was incorporated at 1 mol% of total lipid in the organic phase. The ionizable lipid to total nucleic acids mass ratio was 10:1 and 8:1 w/w for in vitro and in vivo studies, respectively. mCas9 to sgRNA weight ratio was kept 2:1 w/w at all the conditions.

### Physiochemical Characterization

LNPs encapsulating mCas9 and sgRNA were characterized for particle size (ζ‐average), polydispersity index (PDI), and surface charge (ζ‐potential) using dynamic light scattering (DLS). Briefly, 10 µL of LNPs were diluted in 1 mL of 15 times diluted 1X PBS and transferred to disposable folded capillary cells. The ζ‐average, PDI, and ζ‐potential measurements were obtained from 3 independent formulations and were expressed as mean ± standard deviation (SD). All measurements were recorded at 25 °C using Nanosizer ZS Series (Malvern Instruments, USA).

### Encapsulation Efficiency (%EE) Determination

Encapsulated RNA (mCas9 and sgRNA) were quantified using Quant‐iT RiboGreen assay according to the manufacturers’ protocol and as previously described.^[^ ^46^ ^]^ The solutions used for the RiboGreen assay were diluted in RNase‐free water and filter tips were used throughout the assay. To quantify free RNA and total RNA, 4 µL of LNPs were diluted with 46 µL of PBS or 1% Triton‐X in PBS (1: 12.5 v/v) respectively, and incubated at room temperature (RT) under gentle shaking for 20 min. Each sample was then transferred to a black 96‐well plate containing 50 µL of Quant‐iT RiboGreen RNA Reagent (1:500 v/v in PBS). Fluorescence intensity of intact and disrupted LNPs samples was measured using FLUO star OPTIMA plate reader (BMG Labtech, DE) at λex/λem 485/530 nm and 1000 gain. Encapsulated RNA was quantified by subtracting free (unencapsulated) nucleic acids amount from the total added using the following equation:

(1) $$EE\left(\%\right)=\left(\frac{total-free}{total}\right)\times 100$$

### Polymerase Chain Reaction (PCR)

To amplify the target regions of PD‐L1 and CD47 genes (Table S2, Supporting Information), DNA was extracted from NPE‐IE cells using MasterPure Complete DNA & RNA Purification Kit following the manufacturer's instructions. Forward and reverse primers were designed and purchased from Integrated DNA Technologies (IDT) (Table S3, Supporting Information). PD‐L1 and CD47 PCR amplicons were generated via PCR amplification using OneTaq Hot Start 2X Master Mix following the manufacturer's instructions. Briefly, for a 15 µL reaction, OneTaq Hot Start 2X Master Mix (7.5 µL), primers (3 µL, 1 µm), and template DNA (1.5 µL, 10 ng µL^−1^) were added to a PCR tube and mixed. The reaction mixture was then incubated in a T100 Thermal Cycler (Bio‐Rad, USA) following the settings shown in Table S4 (Supporting Information). PCR products were purified using Monarch PCR and DNA Cleanup Kit and quantified using NanoDrop One/OneC Microvolume UV–vis Spectrophotometer (Thermo Fisher Scientific, UK). The purity of the PCR amplicons was verified by agarose gel electrophoresis (1.5% w/v) in sodium borate buffer (10 mm sodium hydroxide and 36.5 mm boric acid (pH 8), SB) at 225 V for 40 min (15 V/cm). Bands were visualized using the ChemiDoc MP system (Bio‐Rad, USA).

### In Vitro Nuclease Assay

An in vitro nuclease assay was used to validate the targeting ability of the designed sgRNAs against PD‐L1 and CD47 genes (Table S2, Supporting Information). First, RNP complexes were prepared following the incubation of Cas9 nuclease (1 µL, 1 µm) and targeting sgRNAs (3 µL, 300 nm) in 1x Cas9 nuclease reaction buffer (23 µL) for 10 min at RT. A non‐targeting sequence was used as a negative control. PD‐L1 or CD47 DNA amplicons (3 µL, 30 nm) generated by PCR were added to the RNP complex and incubated for 15/30/45 min at 37 °C. Final concentrations of Cas9, sgRNA, and DNA were kept at 30, 30, and 3 nm, respectively. The enzymatic reaction was stopped using 6× gel loading dye (6 µL), containing 10 mm EDTA acting as a metal‐chelator for magnesium, thus inhibiting the enzymatic activity of Cas9 nuclease. Digested amplicons were resolved on a native 1.5% w/v SB agarose gel and visualized using the ChemiDoc MP system.

### Cell Culture

Mouse (murine) NPE‐IE glioblastoma stem cells (GSCs) were produced as previously described^[^ ^39^ ^]^ (Scheme S1, Supporting Information). Briefly, Neuronal stem cells (NSC) were extracted from C57BL/6 mice, subjected to neurofibromatosis type 1 (Nf1) and phosphatase and tensin homolog (Pten) deletions, followed by the insertion of epidermal growth factor receptor variant III (EGFRvIII), luciferase (Luc) and green fluorescent protein (GFP). The so‐called NPE cells were passaged once in immune‐competent BL6 mice producing tumor‐derived (TD) NPE cells or NPE‐BL6‐TD cells. The latter were passaged once in immune‐competent BL6 mice to obtain the more aggressive and immune evasive (IE) NPE or so‐called NPE‐IE cells (Table S5, Supporting Information).

NPE‐IE cells were maintained in DMEM/HAMS‐F12 media containing 1.5 mg mL^−1^ glucose, 1% MEM NEAA, 1% P/S, 1% B‐27, 0.5% N‐2, 0.012% BSA, and 100 µm of 50 mm 2‐mercaptoethanol. Prior to cell seeding, laminin, mouse EGF, and human FGF were freshly added to the complete media to a final concentration of 200/10/10 ng mL^−1^, respectively. Cells were detached using 1 mL of accurate for 3 min at RT and subcultured twice a week at a ratio of 1 to 10 once confluency reached 90%.

Mouse GL261 glioma cells expressing the Red‐Fluc luciferase gene from Luciola Italica (Red‐Fluc) were purchased from Perkin‐Elmer (USA) and cultured in Advanced RPMI 1640 media supplemented with 10% FCS, 1% P/S, 1% GlutaMAX and 1% MEM Non‐Essential Amino Acids. Cells were trypsinized with 0.05% trypsin‐EDTA when they reached 90% confluency and were passaged every 3–4 days. All cells were cultured at 37 °C in a humid environment with a 5% level of CO_2_.

### In Vitro Transfection

For in vitro transfection studies, NPE‐IE and GL261 cells were seeded overnight in a 24‐well plate at a density of 40 000 and 75 000 cells per well, respectively. A volume of LNPs containing 0.5 or 1 µg of total nucleic acids was diluted in 500 µL (1 or 2 µg mL^−1^) of freshly prepared media and added to the cells. Cells were incubated at 37 °C, 5% CO_2_ for a further 48 h. Cells were then transferred into a 12‐well plate for an additional 5 days yielding a total of 7 days transfection. in vitro, gene‐editing was assessed on days 2 and/or 7 by T7 endonuclease mismatch assay and flow cytometry as described below.

### T7 Endonuclease Mismatch Assay

DNA was extracted from cells, using MasterPure Complete DNA and RNA Purification Kit following the manufacturer's instructions. Briefly, the cell pellet was resuspended in 300 µL tissue and cell lysis solution and incubated on ice for 5 min. MPC protein precipitation reagent (150 µL) was added to the lysed sample, vortexed for 10s, followed by centrifugation at 16 000 x g for 5 min at 4 °C. The supernatant was transferred to a clean microfuge tube. Isopropanol (500 µL) was added to the supernatant and mixed by inversion 30–40 times. The mixture was centrifuged at 16 000 x g for 5 min at 4 °C. The supernatant was discarded, and the DNA pellet was rinsed twice with 70% ethanol. Residual ethanol was discarded, and the DNA pellet was resuspended in TE buffer. PD‐L1 or CD47 sgRNA target regions were amplified using PCR, as described above. Amplicons (5 µL) were mixed with 2 µL 10× NEBuffer 2 and 12 µL nuclease‐free water. The amplicons were denatured and reannealed to form heteroduplexes (Table S6, Supporting Information). T7 Endonuclease I (1 µL) was added to the mixture and incubated for 15 min at 37 °C. Proteinase K (1 µL) was added and incubated for 5 min at 37 °C to inactivate T7 Endonuclease I. Digested DNA products were resolved on a 1.5% agarose gel in 10 mm SB at 225 V for 40 min. Bands were quantified using Image Lab Software. The percentage of insertions‐deletions (indel) formation (%) was calculated using the following formula:

(2) $$Indel\;formation\left(\%\right)\;=\;\left(\frac{\sum \;cleaved\;product}{\sum \;cleaved\;product,\;full\;length\;amplicons}\right)\times 100$$

### Flow Cytometry of In Vitro Samples

Cells were harvested following accurate or trypsin treatment and centrifugation at 600 x g for 3 min and then washed twice with PBS. Cells were resuspended in 50 µL primary antibodies against PD‐L1 and CD47 or respective isotypes at 1:200 dilution for 20 min at RT. Cells were then washed twice with PBS and resuspended in 200 µL for flow cytometry acquisition using BD FACSCelesta (BD Biosciences, USA). Data were analyzed using FlowJo software (Treestar, USA). Transfection efficiency is expressed as the percentage of cells that lost the expression of the target gene and/or the reduction in median fluorescence intensity (MFI) compared to untransfected controls. Results were analyzed using one‐way ANOVA. Due to the absence of PD‐L1 expression at the protein level in in vitro cultured NPE‐IE cells, PD‐L1 protein knock‐out was assessed in GL261 cells.

### Establishment of Brain Tumor Models

All animal experiments were conducted in agreement with the project (PBE6EB195, PP8950634) and personal licenses granted by the UK Home Office and in accordance with the UKCCCR Guidelines (1998). Animals were purchased from Charles River (UK) or bred at King's College London at Franklin‐Wilkins Biological Services Unit. Animals were maintained at 22 °C on a 12 h light and dark cycle in Individually Ventilated Cages System (IVCS) with ad libitum access to food and water. All animals were kept in specific pathogen‐free conditions. To establish orthotopic tumor models for NPE‐IE and GL261 cells, 5–6 weeks old female (18–22 g) or male (24–28 g) C57BL/6 mice were anesthetized using isoflurane, the scalp was swabbed with antiseptic, and a midline incision was performed. A hole was drilled through the skull at the following coordinates: 0.5 mm anterior and 1.5 mm to the left of the bregma. Murine NPE‐IE cells (400 000 cells in 2 µL) or GL261 (200 000 cells in 2 µL) were stereotactically (Harvard Apparatus, USA) inoculated (0.2 µL min^−1^) using a Hamilton (Thermo Fischer Scientific, UK) 5 µL syringe at a depth of 2.4 mm. During surgery, mice received a subcutaneous injection (s.c.) of 0.3 mg kg^−1^ of Vetergesic as an analgesic. Subsequently, the incision was sutured, and surgical adhesive was applied. Animals were kept under observation on a heating mat pad until fully recovered from anesthesia. Tumor growth was monitored by bioluminescence imaging (BLI), and mice were sacrificed when signals were in the range of 10^9^–10^10^ or weight loss exceeded 20%. Anesthetized mice received a subcutaneous injection of D‐luciferin (150 mg kg^−1^) and were imaged at 10 (GL261) or 12 (NPE‐IE) min after injection using IVIS Lumina Series III In Vivo Imaging System (Caliper Life Sciences, Perkin Elmer, USA). Tumor growth was expressed as total flux (photons/s) over days. NPE‐IE tumor development occurred on average in 30% of mice following implantation, consistent with the nature of this model,^[^ ^39^ ^]^ as such, group sizes for each study were determined by the number of available mice.

### Intracranial Administration of LNPs

Approximately 2–5 µL of LNPs encapsulating 15 µg of total RNA (0.75 mg kg^−1^) were administered intracranially (i.c.) using a Hamilton syringe at the following coordinates: 0.5 mm anterior and 1.5 mm to the left of the bregma line and at a 2.4 mm depth. Mice received the same intra and post‐operative care adopted for the tumor inoculation. For GL261 and NPE‐IE models, tumor were ready for LNPs administration from days 7 and 10 post‐tumor inoculation, respectively.

### Brain and Cellular Distribution Studies

LNPs labeled with far‐red fluorescent dye DiD (LNPs_DiD_, *n* = 3) at 3 µg dye dose equivalent to 15 µg of total nucleic acids. Twenty‐four hours post LNPs administration, mice were perfused as described below, brains were harvested and imaged for luciferase and GFP expression (λex = 480 nm and λem = 520 nm) and DiD (λex = 620 and λem = 670 nm) using IVIS Lumina Series III In Vivo Imaging System. Fluorescence values for individual brain tissues were obtained by manually drawing regions of interest (ROI) around each brain/brain section. Data are expressed as the average radiant efficiency signals ([p/s]/[µW cm^−2^]). For cellular distribution studies, tumors were dissociated into single‐cell suspension and processed for flow cytometry analysis as described below. Data are presented as DiD+ cells as a percentage of viable cells, or per mg of tumor tissues, or as an MFI of the relevant cell population.

### Preparation of Tumor Single‐Cell Suspensions

Prior preparation of single‐cell suspensions for flow cytometry analysis, mice were perfused to remove circulating immune cells or LNPs in the blood circulation. Mice were sedated by intraperitoneal (i.p.) injection of 0.2 mg kg^−1^ of pentobarbital and underwent cardiac perfusion with 20 mL of 0.9% saline solution. Tumor tissues were removed from the rest of the brain using a scalpel and enzymatically digested to obtain a single‐cell suspension.^[^ ^70^ ^]^ Tissues were minced using scalpels. Minced tissues were then resuspended in RPMI media containing collagenase I (1 mg mL^−1^) and deoxyribonuclease (DNase) I (0.1 mg mL^−1^) and incubated at 37 °C for 1 h under vigorous shaking at 500 rpm (SciQuip, UK). Released cells were passed through a 70 µm cell strainer and washed twice with PBS before staining for flow cytometry analysis.

### Flow Cytometry Analysis of Brain Tumors

Single‐cell suspension was stained with viability marker Zombie Aqua following the manufacturer's protocol. For LNPs uptake studies, single‐cell suspension was stained for fluorescently labeled CD45, F4/80, CD11c, and CD3 antibodies. LNPs uptake in the different cell populations was expressed as percentage positive cells and MFI of DiD signals. For immune phenotyping studies, surface staining was performed using the following antibodies: Pacific Blue anti‐mouse CD45, PerCP/Cyanine5.5 anti‐mouse CD11b, PE anti‐mouse F4/80, BV650 anti‐mouse CD11c, APC/Cyanine7 anti‐mouse Ly‐6G, BV785 anti‐mouse CD4, PE anti‐mouse CD8, PerCP/Cyanine 5.5 anti‐mouse CD3, APC anti‐mouse CD47, PE anti‐mouse PD‐L1. All antibodies were used at a 1:200 dilution except for CD3, which was used at a 1:100 dilution. Cells were fixed with 4% PFA for 20 min at room temperature, then washed twice with PBS. Precision Count Beads (20 µL) were freshly added to the stained cells (200 µL) prior to acquisition on BD FACSCelesta flow cytometer.

Data were analyzed using FlowJo software. Tumor cells were identified by the expression of GFP (CD45^−^/GFP^+^). Immune cells subpopulations (CD45^+^) were identified via staining of surface markers: granulocyte‐like myeloid‐derived suppressor cells (G‐MDSC) (CD45^+^/CD11b^+^/Ly6G^+^), tumor‐associated macrophages/microglia (TAMs) (CD45^+^/CD11b^+^/Ly6G^−^/F4/80^+^), mononuclear‐like myeloid‐derived suppressor cells (M‐MDSC) (CD45^+^/CD11b^+^/Ly6G^−^/F4/80^−^/CD11c^−^/Ly6C^+^), dendritic cells (DCs) (CD45^+^/CD11b^+^/Ly6G^−^/F4/80^−^ /CD11c^+^/Ly6C expression variable), CD4^+^ T cells (CD45^+^/CD3^+^/CD4^+^) and CD8^+^ T cells (CD45^+^/CD3^+^/CD8^+^) cells. Results are expressed as a percentage of CD45^+^ cells or absolute cell counts (cells mg^−1^ of tumor). PD‐L1 and CD47 expression is presented as MFI value or fold increase relative to FMO.

### Inference of CRISPR Edits (ICE) Analysis

Tumor‐free or GL261/NPE‐IE tumor‐bearing C57BL/6 mice, receiving either LNPs targeting PD‐L1 (LNPs_sgPD‐L1_) or CD47 (LNPs_sgCD47_) were sacrificed 2, 7, and 10 days post‐LNPs injection (*n* = 3–4 per group). DNA was extracted from brain/tumor tissues using TRIzol reagent, following the manufacturer's instructions. PD‐L1 or CD47 PCR amplicons (5 µL, 10–50 µg µL^−1^) were pre‐mixed with target‐specific reverse primer (5 µL, 5 µm) as reported in Table S7 (Supporting Information) and sent to Genewiz/Azenta for Sanger sequencing. Sanger trace data of control and treated samples (.ab1 files) were uploaded on Inference of CRISPR Edits (ICE) software (https://ice.synthego.com/)^[^ ^71^ ^]^ and reported as percentage of gene knock‐out, distribution of indel frequency or dominant indels. Results were analyzed using one‐way ANOVA.

### Assessment of In Vivo Immunomodulatory Activity of LNPs in NPE‐IE Tumor Model by Flow Cytometry

NPE‐IE tumor‐bearing C57BL/6 mice received a single *i.c*. injection of LNPs encapsulating dual targeting sgRNAs against PD‐L1 and CD47 (LNPs_sgPD‐L1/sgCD47_, *n* = 8), or non‐targeting sgRNA (LNPs_sgNeg_, *n* = 6). One group of animals (*n* = 5) was left untreated. After 10 days post‐LNPs injection, animals were sacrificed to assess changes in PD‐L1 and CD47 expression, as well as alterations in the immune cells profile within the tumor microenvironment using flow cytometry analysis as described above. Results are expressed as cell count per mg of tumor. PD‐L1 and CD47 expression is presented as MFI value.

### Tumor Growth Delay Studies

C57BL/6 mice bearing NPE‐IE tumors were either left untreated (*n* = 9) or received a single i.c. injection of either LNPs_sgPD‐L1_ (*n* = 5), or LNPs_sgCD47_ (*n* = 5), or LNPs_sgNeg_ (*n* = 9). Tumor growth was measured via BLI on days 7, 10, 14, 17, and 20 post‐tumor inoculation. Data were expressed as total flux (photons/s) over days.

### Statistical Analysis

Numerical data were analyzed using GraphPad Prism 9 (La Jolla, CA). Unless otherwise specified, results were analyzed using Student's *t*‐test, as follows. The normality of the data was first assessed using the Shapiro‐Wilks test. Depending on the normality outcome, the Student's *t*‐test was conducted with or without Mann–Whitney post‐test. Statistical significance is indicated by ^*^ *p* < 0.05, ^**^ *p* < 0.01, ^***^ *p* < 0.001 and, ^****^ *p* < 0.0001.

## Conflict of Interest

The authors declare no conflict of interest.

## Author Contributions

N.R., S.M.P., and K.A.J. conceptualized the project. N.R., Y.M.L., J.E.A., A.A.W., J.N.A., and J.W. performed methodology. N.R., A.Z., Y.M.L., A.M., and R.L.O. performed the investigation. N.R., J.E.A., A.A.W., and J.N.A. performed data visualization. J.W. and K.A.J. provided supervision. S.M.P. and K.A.J. provided the resources. K.A.J. acquired the fundings. N.R. wrote the original draft. N.R., A.A.W., and K.A.J. performed writing—review & editing.

## Supporting information



Supporting Information

## Data Availability

The data that support the findings of this study are available from the corresponding author upon reasonable request.
