# Supplementary material for: CD47 Knock‐Out Using CRISPR‐Cas9 RNA Lipid Nanocarriers Results in Reduced Mesenchymal Glioblastoma Growth In Vivo
Source: Adv Sci (Weinh). 2025 Jan 31;12(12):2407262. doi: 10.1002/advs.202407262 (PMC11948039; doi:10.1002/advs.202407262)
Supplement: Supplementary file 1 — Supporting Information [file ADVS-12-2407262-s001.docx]

***Advanced Science***

**Manuscript Submission**

Supplementary Materials for

**CD47 knock-out using CRISPR-Cas9 RNA lipid nanocarriers results in reduced mesenchymal glioblastoma growth *in vivo***

Nadia Rouatbi *et al.*

* Corresponding author

E-mail: [khuloud.al-jamal@kcl.ac.uk](mailto:khuloud.al-jamal@kcl.ac.uk)

**This PDF file includes:**

Supplementary Results

Figs. S1 to S9

Tables S1 to S7

Scheme S1

Supplementary Results

**Orthotopic NPE-IE and GL261 tumor growth characterization**

In the current study, orthotopic GL261 and NPE-IE tumor models were established through stereotactic injection of cancer cells into the left hemisphere of C57BL/6 hosts, as outlined in the methodology section. As shown in **Figure S3**, tumors were detected as early as day 3 in both models. Notably, in the GL261 model, tumor formation was observed in all inoculated animals, regardless of whether C57BL/6 hosts were obtained from Charles Rivers (**Figure S3A**) or bred internally at the university’s facility (**Figure S3B**). GL261 tumors were able to grow until day 19, reaching an average size that produces bioluminescence signals of 7.43E^8^ (**Figure S3A**). Conversely, in the NPE-IE model, between days 3 and 10, some of the inoculated mice exhibited tumor rejection, leading to a decrease in the success rate of tumor establishment. By day 10, NPE-IE tumor formation rates were ~39% in C57BL/6 hosts from Charles River (**Figure S3C**) and ~19% in C57BL/6 hosts bred internally (**Figure S3D**). This tumor rejection in the NPE-IE model was as anticipated, albeit with a relatively higher incidence, in line with the findings of Gangoso *et al.* *Cell. 2021*.

**Table S1.**

LNPs composition.

| **LNPs formulation (5 µg RNA, NP 5)** | | | | |
| --- | --- | --- | --- | --- |
| **Lipid Phase** | **Concentration (mg/mL)** | **Amount (µg)** | **Amount (µL)** | **Amount (nmol)** |
| Dlin-MC3-DMA^*^ | 2 | 50 | 25 | 77.86 |
| Cholesterol | 2 | 40 | 20 | 103.45 |
| DOPE^†^ | 2 | 26.5 | 13.25 | 35.58 |
| C16 PEG 2000^‡^ | 4 | 14 | 3.5 | 5.55 |
| Citrate buffer^§^ |  |  | 6.19 |  |
| **Water Phase** | **Concentration (mg/mL)** | **Amount (µg)** | **Amount (µL)** | **Amount (nmol)** |
| mCas9 | 1 | 3.3 | 3.3 | 0.0022304 |
| sgRNA | 1 | 1.7 | 1.7 | 0.051345 |
| Citrate buffer |  |  | 95 |  |

^*^ Dilinoleylmethyl-4-dimethylaminobutyrate

^†^ 1, 2 dioleoyl-sn-glycero-3-phosphoethanolamine

^‡^ N-palmitoyl-sphingosine-1-[succinyl(methoxy polyethylene glycol) 2000]

^§^ For LNPs for *in vivo* tracking contained DiD at 1% mol of total lipid

**Table S2.**

sgRNA target sequences.

| **Target gene** | **Target sequence** | **Genomic location** | **GC (%)** | **MM0** | **MM1** | **MM2** | **MM3** |
| --- | --- | --- | --- | --- | --- | --- | --- |
| Mouse PD-L1 | GCTTGCGTTAGTGGTGTACTGGG | chr19:29351055 | 50 | 0 | 0 | 0 | 0 |
| Mouse CD47 | CCCTTGCATCGTCCGTAATGTGG | chr16:49688198 | 55 | 0 | 0 | 0 | 1 |

**Table S3.**

PCR forward (F) and reverse (R) primers used for target-specific amplicons generation.

| **Purpose** | **Primer** | **Sequence** | **T_m_ (^°^C)** | **Amplicon size (bp)** |
| --- | --- | --- | --- | --- |
| Nuclease/T7 mismatch assays | PD-L1 F | AGTTACTTGGAAAGGATGAAGGA | 62 | 970 |
|  | PD-L1 R | GAAGTGTGTGAACGAACGAATG |  |  |
| Sanger sequencing | PD-L1 F | TAACAGGTGATCCGTTTCCTATG | 62 | 598 |
|  | PD-L1 R | GCACCACCGTAGCTGATTAT |  |  |
| Nuclease/T7 mismatch assays & Sanger sequencing | CD47 F | TTGGGTTATGCAGCCTGAGT | 62 | 929 |
|  | CD47 R | CACTTGCCCAAGAAGAGCCT |  |  |

**Table S4.**

Conditions for PCR reaction.

| **Cycle step** | **Temperature (°C)** | **Time** | **Number of cycles** |
| --- | --- | --- | --- |
| Initial denaturation | 94 | 5 min | 1 |
| Denaturation | 94 | 30 sec | 40 |
| Annealing | Tm | 1 min | 40 |
| Extension | 68 | 1 min | 40 |
| Final extension | 68 | 5 min | 1 |

**Scheme S1.**

Schematic representation of the engineering of NPE-IE glioblastoma stem cells. Created with Biorender.com.


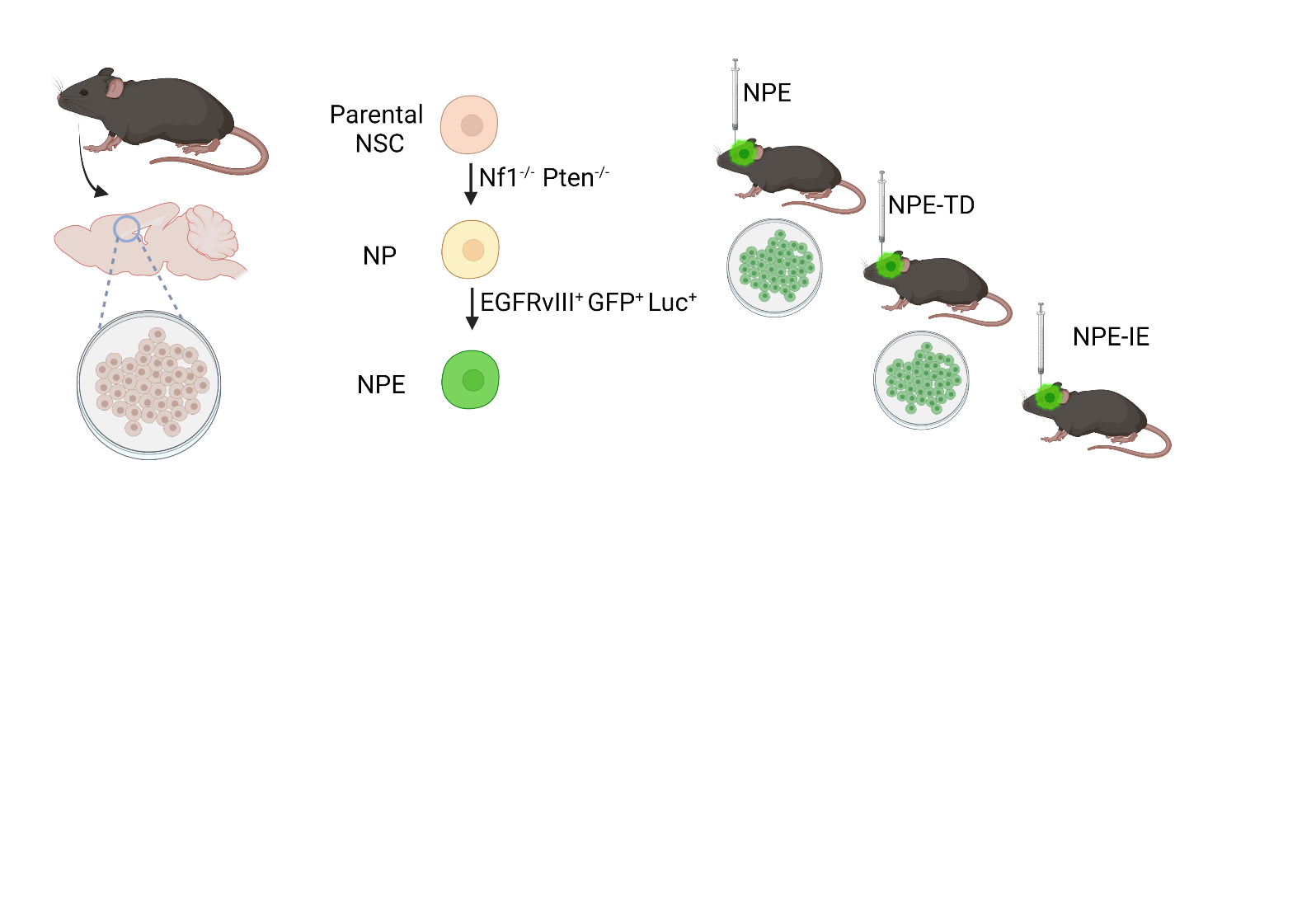


**Table S5.**

Characteristics of NPE-IE glioblastoma stem cell line.

| **Primer** | **NPE-IE** |
| --- | --- |
| **Tumor model** | GSCs |
| **Subtype** | Mesenchymal |
| **Species of origin** | C57BL/6 J |
| **Cell of origin** | Neural stem cells |
| **Genetic deletions** | Nf1, Pten |
| **Genetic insertions** | EGFRvIII |
| **Reporter genes** | GFP, Luc |
| **Immune target expression** | CD47 (*in vitro* and *in vivo*)  PD-L1 (*in vivo* only) |
| **Immune evasion capabilities** | High |
| **Intratumoral immune cell infiltration** | High |

**Table S6.**

Conditions for heteroduplex formation.

| **Cycle step** | **Temperature** | **Ramp rate** | **Duration** | |
| --- | --- | --- | --- | --- |
| Denaturing | 95 °C |  | 5 min | |
| Annealing | 95 – 85 °C | -2 °C/s | |  |
|  | 85 – 25 °C | -0.1 °C/s |  | |
| Hold | 4 °C |  |  | |

**Table S7.**

Reverse (R) primer used for Sanger Sequencing.

| **Purpose** | **Primer** | **Sequence** |
| --- | --- | --- |
| Sanger Sequencing | PD-L1 R | ACCACCGTAGCTGATTATGC |
| Sanger Sequencing | CD47 R | CACTTGCCCAAGAAGAGCCT |

**Fig. S1.**

**Both mCas9 and sgRNA are loaded into LNPs.** LNPs formulated to encapsulate mCas9 and sgRNA were exposed to RNase A to digest any free RNA. RNase A was inactivated by proteinase K/heating. LNPs were disassembled with Heparin (10% v/v) and the released nucleic acids were purified by Monarch RNA Cleanup Kit and resolved on 2% denaturing agarose gel and imaged using ChemiDoc™ MP system. Agarose gel electrophoresis of mCas9, sgRNA, mixture of mCas9 and sgRNA (mix), and mCas9/sgRNA extracted from the LNPs. The intensity ratio between mCas9 and sgRNA was calculated on the intensity values obtained using Image Lab software.


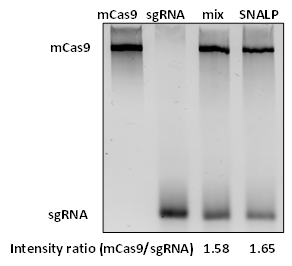


**Fig. S2.**

**LNPs-mediate inefficient *in vitro* gene editing of PD-L1 and CD47 in GL261 cells.** Briefly, GL261 cells were transfected with LNPs for 48 h at a concentration of 2 µg/mL of nucleic acids composed of Cas9 mRNA (mCas9) and a negative sgRNA (Negative) or targeting sgRNA against PD-L1 or CD47. Gene editing was quantified using Interference of CRISPR Edits (ICE) analysis on DNA samples extracted 7 days post-transfection. Percentage gene knock-out (KO) calculated using ICE analysis on Sanger Sequencing traces **(A)**. Protein knock-out measured at 2- and 7- days post-transfection using flow cytometry and expressed as percentage PD-L1-positive (PD-L1^+^) **(B)** and CD47-positive (CD47^+^) **(D)**. Median fluorescence intensity (MFI) of PD-L1 **(C)** and CD47 **(E)** expression calculated on the entire cell population at 2- and 7-days post-transfection. Results are expressed as means ± SD, (n = 3).


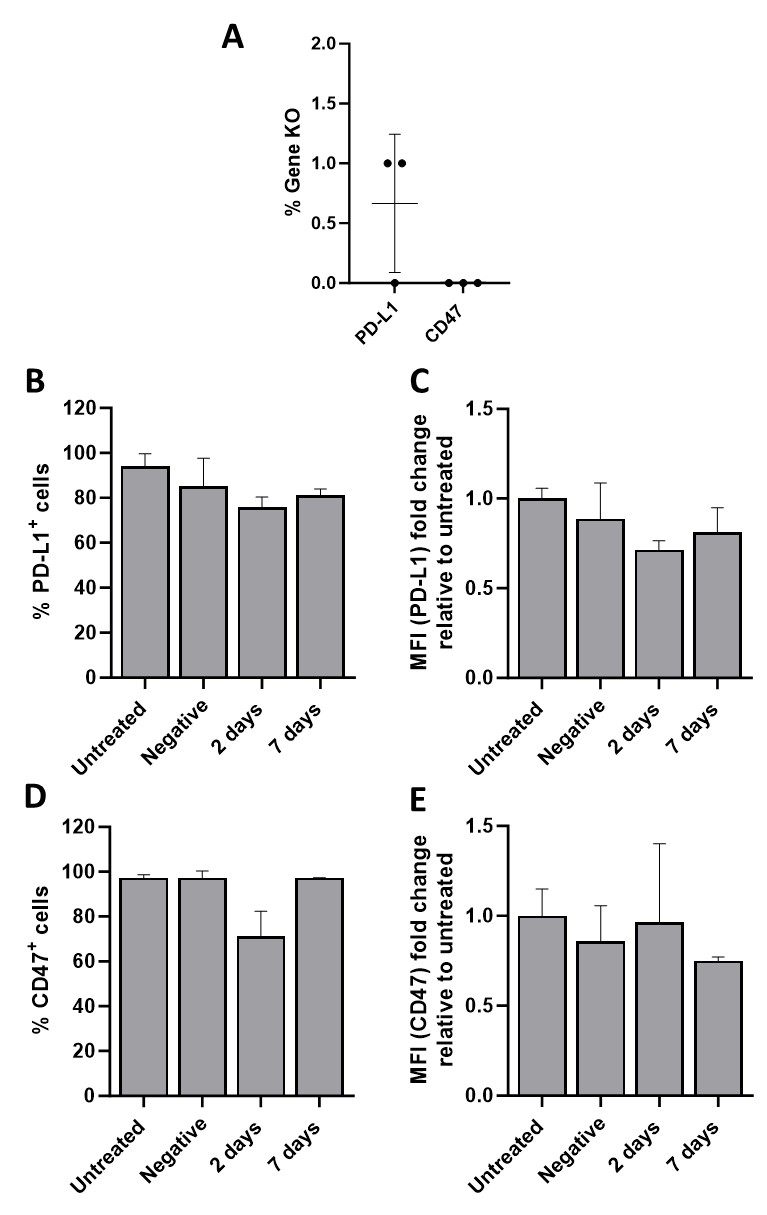


**Fig. S3.**

**Summary of tumor growth curves for GL261 and NPE-IE tumor models.** Gl261 (200K) or NPE-IE (400K) cells were implanted intracranially (*i.c.*) in the brains of female or male C57BL/6 mice. Tumor growth curves were obtained by whole-body Bioluminescence Imaging (BLI) using IVIS Lumina III system and are expressed as Bioluminescence Intensity (BLI, photons/seconds) over time. Tumor growth of GL261 tumors **(A, B)**. Tumor growth of NPE-IE tumors **(C, D)**. Animals were purchased from Charles River **(A, C)**. Animals were bred internally **(B, D)**.


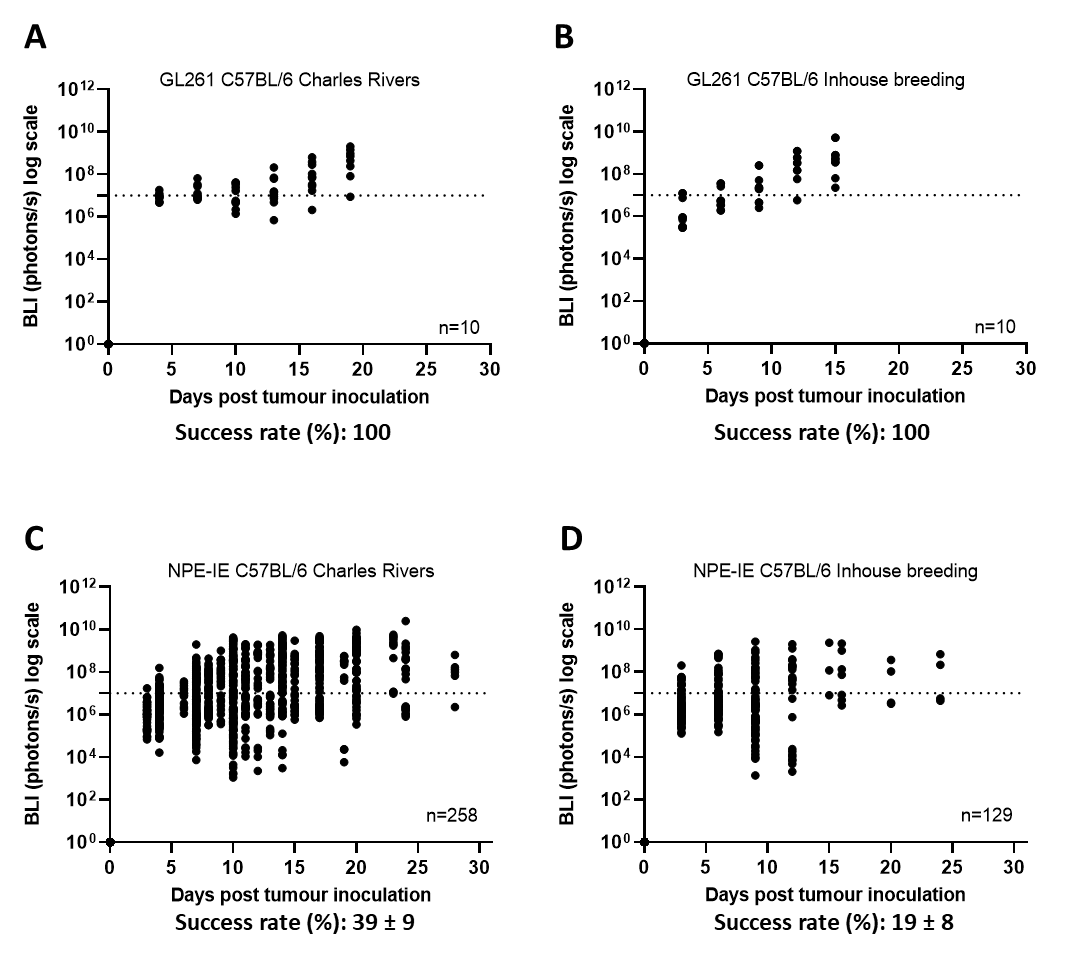


**Fig. S4.**

**Intracranial injection of LNPs can efficiently mediate indel formation around PD-L1 and CD47 cleavage sites in tumor-free brain parenchymal tissue as well as GL261 and NPE-IE orthotopic tumors.** Briefly, LNPs encapsulating mCas9 and targeting sgRNA against PD-L1 (LNPs_sgPD-L1_) or CD47 (LNPs_sgCD47_) were (*i.c.*) injected into tumor-free brain parenchymal tissue or GL261/NPE-IE tumors, at a nucleic acid dose of 15 µg. Two, seven- and ten-days post-injection animals were sacrificed, brain/tumoros tissues were digested, and DNA was extracted for PCR amplification, Sanger Sequencing and Interference of CRISPR Edits (ICE) analysis. Combined (day 2 and 7) indel formation pattern for PD-L1 **(A)** and CD47 **(B)** expressed as the frequency of indel at a specific genomic position relative to the cleavage sites (position = 0).


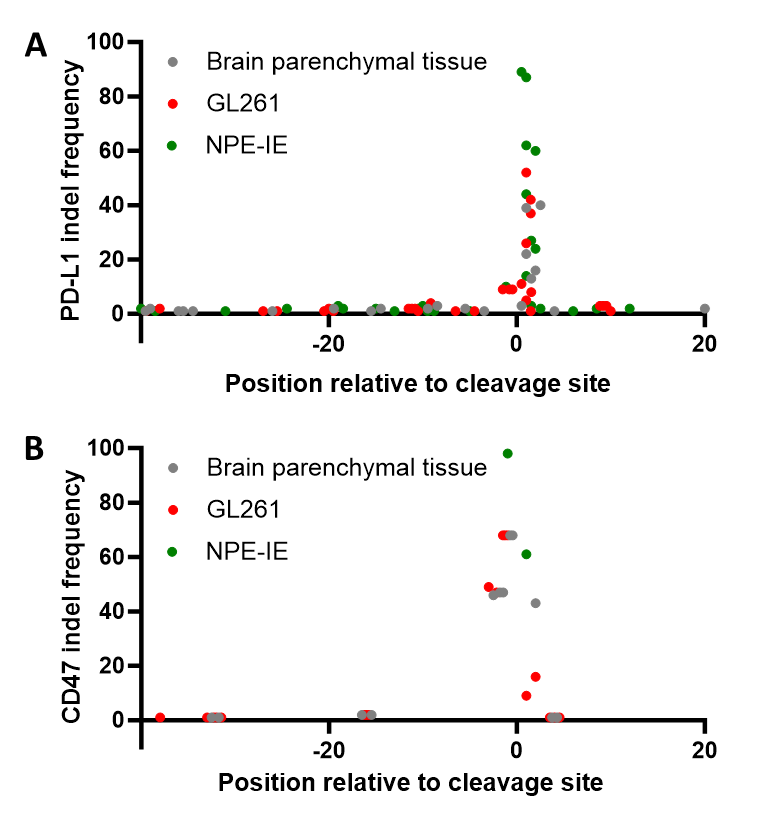


**Fig. S5.**

**Prolonged LNPs accumulation in the brain observed after intracranial administration in orthotopic NPE-IE tumors compared to tumor-free brain parenchymal tissue.** Briefly, LNPs labelled with near infrared dye (DiD) were intracranially (*i.c.*) injected in tumor-free brain parenchymal tissue of C57BL/6 mice (n = 1) or mice bearing orthotopic NPE-IE tumors (n = 1). Twenty-four hours post-injection animals were sacrificed and transcardially perfused with saline. One mouse was left untreated and used as negative control. Brain tissues were imaged using IVIS Lumina III system. Brain/tumor tissue surrounding the area of injection in C2 and C3 were dissected and enzymatically digested to obtain a single-cell suspension, which was assessed for viability and stained for flow cytometry analysis. Representative optical images of brains and brain sections of untreated NPE-IE tumor bearing brain **(A)** and tumor-free brain parenchymal tissue **(B)** injected with LNPs_DiD_. Bioluminescence and fluorescence expression of Luciferase (Luc) and Green Fluorescence Protein (GFP) indicate the presence of the tumor, while DiD signal is representative of the LNPs distribution. Semi-quantitative analysis of the DiD signal present in the whole brain **(C)** and in the coronal sections **(D)** expressed as total radiant intensity. Flow cytometry quantification of LNPs_DiD_ uptake within the brain/tumor tissue surrounding the area of injection in C2 and C3 **(E, F)**. Percentages (%) of DiD+ cells calculated among the viable cells in tumor-free parenchymal tissue and NPE-IE tumors **(E)**. Median fluorescence intensity (MFI) of DiD signal in tumor-free and NPE-IE tumors **(F)**. Results are expressed as means ± SD.


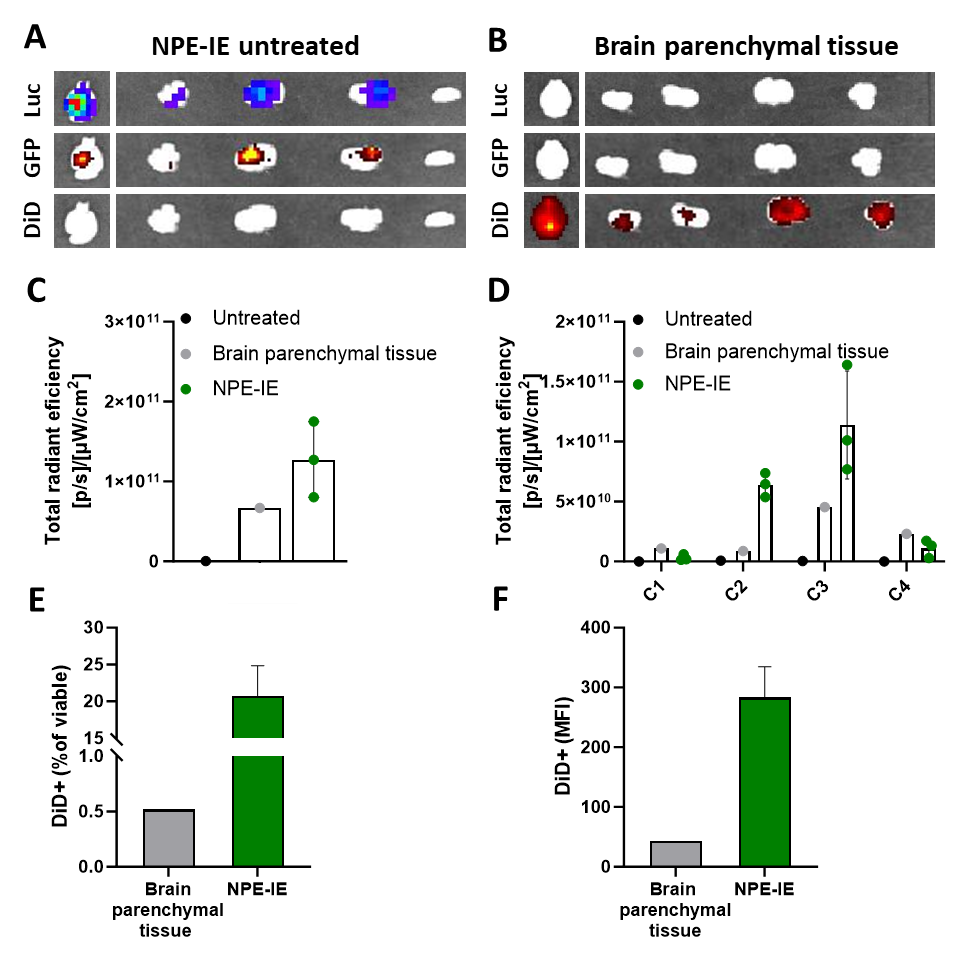


**Fig. S6.**

**Hierarchical multi-parameteric flow cytometry gating strategy for immunophenotyping of orthotopic NPE-IE tumors.** NPE-IE tumors extracted from perfused mouse brains were dissected and enzymatically digested to obtain a single-cell suspension which was assessed for viability and phenotyped using different surface markers **(A)**. First, singlets were selected by gating events in the diagonal FSC-H vs. FSC-A plots. Live cells were gated out from the positively stained population with Zombie Aqua™ viability marker. Identification of tumor infiltrating leukocytes (CD45^+^/GFP^-^) **(B)** and cancer cells (CD45^-^/GFP^+^) **(C)**. From the infiltrating leukocytes the following immune cell populations were identified: granulocyte-like myeloid-derived suppressor cells (G-MDSC) (CD45^+^/CD11b^+^/Ly6G^+^) **(D)**, tumor associated macrophages/microglia (TAMs) (CD45^+^/CD11b^+^/Ly6G^-^/F4/80^+^) **(E)**, mononuclear-like myeloid-derived suppressor cells (M-MDSC) (CD45^+^/CD11b^+^/Ly6G^-^/F4/80^-^/CD11c^-^/Ly6C^+^) **(F)**, dendritic cells (DCs) (CD45^+^/CD11b^+^/Ly6G^-^/F4/80^-^/CD11c^+^/ Ly6C expression variable) **(G)**, CD4^+^ (CD45^+^/CD4^+^) and CD8^+^ (CD45


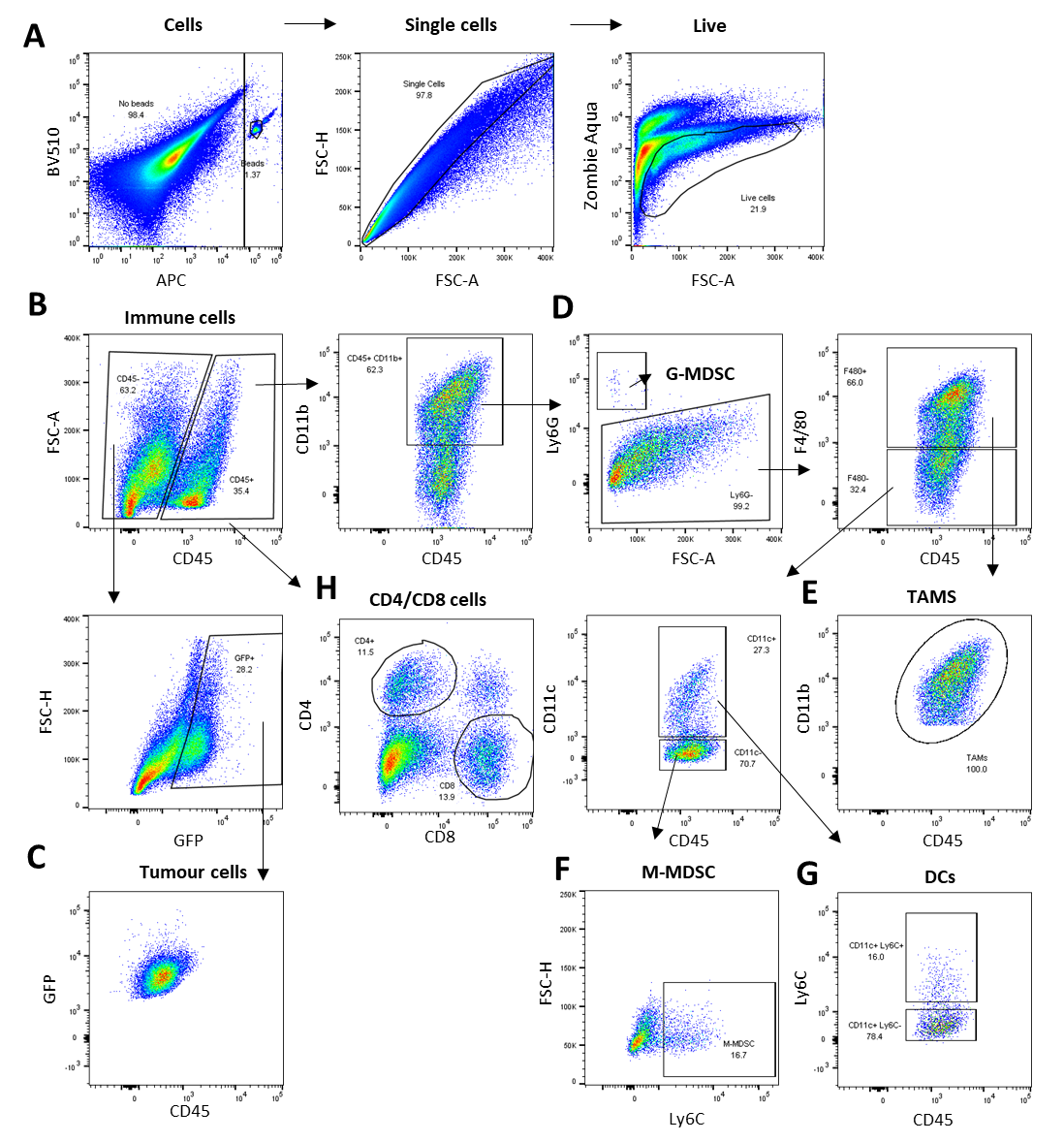


Fig. S7

Immunophenotyping of orthotopic NPE-IE tumors following LNPs intracranial administration. C57BL/6 mice bearing orthotopic NPE-IE tumors were intracranially (i.c.) injected with LNPs encapsulating mCas9 and a combination of targeting sgRNA against PD-L1 and CD47 (PD-L1/CD47, n = 8), or non-targeting sgRNA (Negative, n = 6), at a nucleic acid dose of 15 µg. One group was left untreated (n = 5). Ten days post-injection animals were sacrificed, and tumors extracted from perfused mouse brains were dissected and enzymatically digested to obtain a single-cell suspension, which was assessed for viability and stained with anti-mouse CD45, CD11b, Ly6G, F4/80, CD11c, CD4, CD8. Absolute counts of cancer cells (GFP+) (A), dendritic cells (DCs) (B) expressed as cells per mg of tumor. Ratio between CD4+ and CD8+ cells (CD4+/CD8+) (C). Absolute counts were obtained by including precision counting beads prior to acquisition on the flow cytometer. Results are expressed as means ± SD. Each point represents an individual mouse.


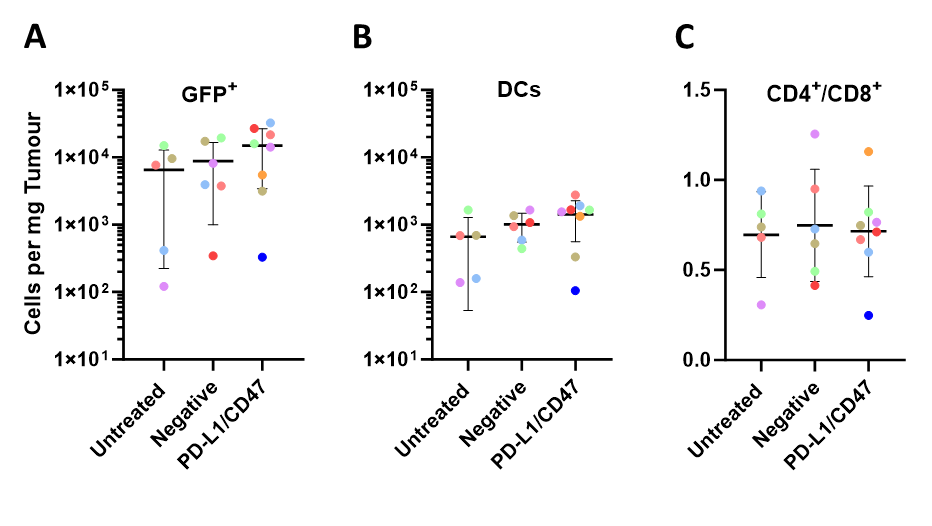


Fig. S8

Percentage weight change of NPE-IE bearing tumors throughout the course of the treatment. C57BL/6 mice bearing orthotopic NPE-IE tumors were intracranially (*i.c.*) injected with LNPs encapsulating mCas9 and targeting sgRNA against PD-L1 (PD-L1, $\boldsymbol{n=5}$), or CD47 (CD47, $\boldsymbol{n=5}$) or non-targeting sgRNA (Negative, $\boldsymbol{n=9}$), at a nucleic acid dose of 15 µg. One group was left untreated ($\boldsymbol{n=8}$). The animals’ weight was monitored throughout the treatment, and changes were expressed as a percentage relative to their initial weight. Data are presented for the the control (A), negative (B), CD47 (C), PD-L1 (D) groups.

Fig. S9

Tumor growth images. Briefly, C57BL/6 mice bearing orthotopic NPE-IE tumors were intracranially (*i.c.*) injected with LNPs encapsulating mCas9 and targeting sgRNA against PD-L1 (PD-L1, $\boldsymbol{n=5}$), or CD47 (CD47, $\boldsymbol{n=5}$) or non-targeting sgRNA (Negative, $\boldsymbol{n=9}$), at a nucleic acid dose of 15 µg. One group was left untreated ($\boldsymbol{n=8}$). Bioluminescence Intensity was measured using IVIS Lumina III system to assess changes in the tumor growth post-LNPs treatment. Representative BLI images of mice at day10 (A), 14 (B) and 17 (C) post tumor inoculation; all images are reported at the same color scale, with min/max radiance of 10^7^/10^9^ photons/sec/cm2/sr.
